# German Society of Neurology guidelines for the diagnosis and treatment of cognitive impairment and affective disorders in people with Parkinson’s disease: new spotlights on diagnostic procedures and non-pharmacological interventions

**DOI:** 10.1007/s00415-024-12503-0

**Published:** 2024-08-09

**Authors:** Elke Kalbe, Ann-Kristin Folkerts, Karsten Witt, Carsten Buhmann, Inga Liepelt-Scarfone, for the German Parkinson’s Guidelines Group

**Affiliations:** 1 Medical Psychology | Neuropsychology and Gender Studies & Center for Neuropsychological Diagnostics and Intervention (CeNDI), Faculty of Medicine and University Hospital Cologne, University of Cologne, Cologne, Germany; 2 Department of Neurology, School of Medicine and Health Science, Carl Von Ossietzky University of Oldenburg, Oldenburg, Germany; 3 Research Center of Neurosensory Science, Carl Von Ossietzky University of Oldenburg, Oldenburg, Germany; 4 Department of Neurology, Evangelical Hospital, Oldenburg, Germany; 5 Department of Neurology, University Clinic Eppendorf, Hamburg, Germany; 6 Hertie Institute for Clinical Brain Research, Department of Neurodegenerative Diseases, Eberhard Karls Universität Tübingen, Tübingen, Germany; 7 German Center for Neurodegenerative Diseases (DZNE), Tübingen, Germany; 8 IB-Hochschule, Stuttgart, Germany

**Keywords:** Mild cognitive impairment, Dementia, Depression, Anxiety, Parkinson’s disease, Guideline

## Abstract

**Background and objective:**

Cognitive impairment and dementia as well as affective disorders are common and debilitating syndromes that develop in people with Parkinson’s disease (PwPD). The authors summarized recommendations for the 2023 updated German guidelines on “Parkinson’s disease” from the German Neurological Society (DGN), focusing on the diagnosis and treatment of these disorders.

**Methods:**

The recommendations were based on literature reviews, other relevant guidelines, and expert opinions.

**Results:**

Measurements to assess cognitive and affective states were reviewed for psychometric properties, use in routine clinical practice, and availability in German. To improve mild cognitive impairment, cognitive training and physical aerobic training are recommended. To treat Parkinson’s disease (PD)-related dementia, cognitive stimulation (as a non-pharmacological intervention) and acetylcholinesterase inhibitors (AChEIs, i.e., rivastigmine) are recommended. Cognitive behavioral therapy is recommended to treat depression, anxiety, and fear of progression. Physical interventions are recommended to treat depression, fatigue, and apathy. Optimized dopaminergic treatment is the first-line pharmacological strategy recommended to manage depression, apathy, anhedonia, fatigue, and mood swings. Major depression can be additionally treated using venlafaxine or desipramine, while moderate depression can be treated pharmacologically according to its clinical phenotype (psychomotor retardation or agitation) and comorbidities (e.g., sleep disturbances, pain). Venlafaxine and nortriptyline can be used to treat anhedonia, while citalopram can be used for anxiety.

**Conclusions:**

In addition to the updated pharmacological treatment options, new insights into recommendations for standardized diagnostics and non-pharmacological interventions were provided for the German health care system. However, more studies are needed to explore the full potential of non-pharmacological interventions to treat and prevent cognitive impairment and affective disorders.

**Supplementary Information:**

The online version contains supplementary material available at 10.1007/s00415-024-12503-0.

## Introduction

In people with Parkinson’s disease (PwPD), cognitive impairment, including Parkinson’s disease mild cognitive impairment (PD–MCI) and Parkinson’s disease dementia (PDD), as well as affective disorders, are the most common and debilitating non-motor symptoms [1–3]. However, affective disorders are still largely underdiagnosed [4]. For the best medical treatment, the standardization of diagnostic and therapeutic strategies is of the utmost importance.

The new German S2k guidelines for “Parkinson’s disease” (PD) provide up-to-date recommendations for the diagnosis and treatment of PwPD in clinical practice [5]. This paper summarized the guidelines’ chapters of “Cognitive impairment” and “Affective disorders.” In the “Cognitive impairment” chapter, both diagnostic and treatment guidelines for PD–MCI and PDD are included, and in the “Affective disorders” chapter, guidelines for the diagnosis and treatment of depression, anhedonia, apathy, anxiety, and fear of progression are included. Fatigue is also considered.

## What is new in the novel S2k guidelines?

Contrary to its previous version, the new S2k guidelines refer to criteria and assessments that can be used to support the diagnosis of cognitive impairment and affective disorders in PwPD in clinical practice. In its recommendations for treatment, both non-pharmacological and pharmacological interventions are considered. The main recommendations are:

### Cognitive disorders

- Proposal of diagnostic criteria and cognitive assessments for PD–MCI and PDD, as well as measures to assess activities of daily living (ADL) to further support the diagnosis of PDD
- Treatment of PD–MCI with cognitive training and physical interventions
- Therapy for PDD with cognitive stimulation, the acetylcholinesterase inhibitor (AChEI) rivastigmine, and the off-label use of donepezil

### Affective disorders

- proposal of medical history questions and scales to assess affective disorders
- treatment of depression, anxiety, and fear of progression with cognitive behavioral therapy (CBT); treatment of depression, apathy, and fatigue with physical interventions
- optimization of dopaminergic medication as a first-line strategy to manage depression, apathy, anhedonia, mood swings, and fatigue
- treatment of depression, anhedonia, apathy, anxiety or panic, and fatigue with non-dopaminergic medication following the results of randomized controlled trials (RCTs), individual antidepressant effect profiles, clinical phenotypes, and comorbidities; treatment with venlafaxine or desipramine for major depression; treatment with venlafaxine, citalopram, or sertraline for moderate depression and coexisting psychomotor retardation; treatment with mirtazapine or trazodone for moderate depression and coexisting agitation; treatment with amitriptyline for moderate depression and comorbid sleep disturbances, hypersalivation, or pain in cognitively intact PwPD; treatment with venlafaxine or nortriptyline for apathy, citalopram for anxiety, and modafinil or safinamide for fatigue.

## Methodology

The new guidelines were prepared as S2k guidelines and ecommendations were based on a structured consensus process. The author team of the chapters dealing with cognitive and affective disorders in PwPD comprised an interdisciplinary group of experts from the fields of neurology (KW, CB), neuropsychology (EK, ILS), and gerontology (AKF). This team developed the key questions that were chosen for the preparation of the recommendations, and the editorial board of the PD guidelines consisting of the publishing association, i.e., the German Neurological Society (DGN), further professional associations and organizations, and experts from the field approved them.

The recommendations were based on (1) literature reviews conducted in the MEDLINE database considering meta-analyses, RCTs, and other relevant studies; (2) German national guidelines that are also relevant for PwPD, i.e., guidelines for the diagnosis and treatment of dementia [6], unipolar depression [7], and anxiety disorders [8]; (3) recommendations from the International Parkinson and Movement Disorder Society (MDS); (4) expert opinions based on clinical expertise and knowledge of the literature; (4) previous versions of the PD guidelines for all questions that were represented therein.

The recommendations followed a “should/should not,” “can/cannot,” or “might/might not” sentence structure. The editorial board of the PD guidelines then discussed and approved the recommendations. Based on the discussions, some revisions were necessary. The final recommendations were then voted on and classified according to their consent of approval rates (strong consent: >95% positive votes; consent: >85–95% positive votes; majority agreement: >50–85% positive votes; no majority agreement: <50% positive votes).

## Cognitive impairment

### Definition, epidemiology, and clinical presentation

Cognitive impairment is one of the most common non-motor symptoms in PwPD [9]. Between 60% and 83% of PwPD progress to PDD during the disease course, especially in more advanced stages [10]. To prevent or delay PDD is urgent, as PDD lowers PwPDs’ health-related quality of life, increases the risk for nursing home placement and mortality, and increases caregiver burden [11]. PD–MCI is considered a possible transitory stage from normal cognition to PDD. Even though not all people with PD–MCI will develop PDD, the presence of PD–MCI, prevalent in 25–30% of PwPD. Therefore, PD-MCI is one of the greatest risk factors for the development of PDD [9, 12], on average, one-third of people with PD–MCI develop dementia within 7 years [13]. Initial evidence has confirmed that the non-pharmacological treatment of people with PD–MCI can enhance or stabilize cognitive function [9]. Older age, male gender, postural instability and gait symptoms, a genetic vulnerability, and concomitant amyloid and tau pathology have also been demonstrated to contribute to the worsening of cognitive function in PwPD [14].

Cognitive impairment in PwPD affects various functions, including executive function, attention, working memory, memory, visuocognitionand language [15]. Besides worsened cognition, loss of the abilities to perform ADL is the core criterion for the diagnosis of PDD [16, 17]. Most importantly, ADL impairment indicative of dementia is primarily caused by cognitive impairment, not by motor or other non-motor dysfunction. The occurrence of behavioral abnormalities (e.g., hallucinations, apathy) further supports the diagnosis of PDD [18].

### Assessment of cognitive impairment

Besides the diagnosis of PD according to consensus guidelines [19, 20], a slow progressive decline in cognitive function, reported either by the PwPD themselves, a third party, or an investigator, within the frame of a long-standing disease course characterizes cognitive impairment in PD [15, 16, 18].

The MDS Task Force [15, 16] recommends two levels of assessment to diagnose PD–MCI and PDD: (1) a short screening for impairment assessed with global cognitive scales and/or impairment in specific cognitive tasks (Level I) and (2) a detailed neuropsychological assessment using at least two tests to evaluate deficits in each of the following domains: executive function, attention, working memory, memory, visuocognition, and language (Level II). Diagnoses of cognitive impairment should be based on assessments with sufficient psychometric properties [15]. Therefore, as a highly novel aspect of these guidelines, it is recommended to use scales available in German to assess global cognition and ADL measures in order to support the diagnosis of cognitive impairment in PD. “*Should be used*” recommendations are to be used if there is sufficient accuracy for diagnosis of cognitive impairment (PD–MCI or PDD) evaluated in international (non-German speaking) and national (German speaking) cohorts, acceptable diagnostic properties (reliability, validity, and sensitivity of change), and if all relevant aspects of impairment are detected by the measurement. “*Can be used*” recommendations are to be used if there is sufficient accuracy for diagnosis of cognitive impairment (PD–MCI or PDD) evaluated in international (non-German speaking) or national (German speaking) cohorts or if cognitive deficits are detected in national cohorts, but incomplete information about diagnostic properties is reported (including reliability, validity, and sensitivity of change) or if not all relevant aspects of (cognitive or ADL) impairment are detected by the measurement. “*Might be used*” recommendations are to be used if sufficient diagnostic accuracy in the assessment of a cognitive impairment diagnosis (PD–MCI or PDD) is evaluated in international (non-German speaking) or national (German speaking) cohorts and if acceptable diagnostic properties in other diseases are given but no information about diagnostic properties in PwPD is available in the German version.

An overview of the diagnostic properties of recommended assessments is displayed in Supplementary Table 1. Scale properties and the diagnostic accuracy of the Montreal Cognitive Assessment (MoCA) for PD–MCI and PDD have been confirmed in international and national cohorts [21–25], and age-, gender-, and education-corrected standard scores are available [26].

The diagnostic properties of the German Mattis Dementia Rating Scale (MDRS) have been validated in international studies, but these properties have not yet been defined in the German version [27]. There is evidence that in the German version of the MDRS, the cognitive performance of PwPD and healthy controls differs greatly (Cohen’s *d*: 0.99 ≤ *d* ≤ 1.01) [28], confirming that the MDRS can detect cognitive impairment in PwPD.

The Parkinson Neuropsychometric Dementia Assessment (PANDA) has been shown to have sufficient reliability and validity for PDD [29], but its diagnostic value has not yet been confirmed for PD–MCI [28]. Additionally, no specific subscale to assess attention is included in the PANDA, and sensitivity of change has not yet been defined in national PD samples [21]. However, since the PANDA includes highly sensitive measures to assess mild cognitive dysfunction in PwPD, it is suggested for use, on the basis of an expert rating, for the Level I diagnosis of PD–MCI.

Scales for Outcomes in Parkinson’s Disease–Cognition (SCOPA–COG) and various versions of the Addenbrooke’s Cognitive Examination (ACE) lack either the confirmation of acceptable scale properties (reliability and sensitivity of change) or the validation of diagnostic accuracy in German cohorts. Further critique of the SCOPA–COG concerns its high weight for memory function on the global scale [21].

Due to its low sensitivity, the Mini Mental State Examination (MMSE) is only recommended for the Level I diagnosis of PDD. The MDS Task Force recommends a cut-off of <26 points for the MMSE [21], but higher (<29 points, sensitivity of 78%, specificity of 63%, [30]) and lower (<25 points, sensitivity of 71%, specificity of 90%, [24]) cut-off scores have also been reported [24, 30]. As a further limitation, the MMSE does not include items to assess executive function [21]. For the German version, age-, gender-, and education-corrected norm values are available and recommended [31].

**Table:** 

*Recommendations for scales to assess cognitive impairment (new)*
Cognitive screening tools should be used for the Level I diagnosis of PD–MCI. Corrections for age, education, and, if necessary, gender should be applied whenever available
The following instruments are particularly suitable (*can be used*)
- MoCA with a cut-off of <26 points
- MDRS with a cut-off of <140 points
The following instruments are also recommended (*might be used*)
- SCOPA–COG with a cut-off of <30 points
- ACE or ACE–R with cut-off values dependent on education
- PANDA with a cut-off of <17 points
Level of consensus: 100%, strong consensus
Cognitive screening tools should be used for the Level I diagnosis of PDD. Corrections for age, education, and, if necessary, gender should be applied whenever available
The following instruments are particularly suitable (*can be used*)
- MoCA with a cut-off of <21 points
- MDRS with a cut-off of <123 points
- MMSE with a cut-off of <26 points
- PANDA with a cut-off of <15 points
The following instruments are also recommended (*might be used*)
- SCOPA–COG with a cut-off of <23 points
- ACE or ACE–R with cut-off values dependent on education
Level of consensus: 100%, strong consensus
For the Level II diagnosis of PD–MCI, the following aspects must be considered:
- For Level II testing, two tests should be used for each of the five cognitive domains (executive function, attention and working memory, memory, language, visuocognition)
- At least two tests of different functions in a cognitive domain should be used to diagnose domain-specific cognitive disorders
- A value in the range of 1–2 standard deviations below the population mean should be used as a cut-off to define cognitive impairment
Level of consensus: 100%, strong consensus
The following aspects must be considered for the Level II diagnosis of PDD:
- To confirm the diagnosis of PDD using Level II testing, two tests should be used for each of the five cognitive domains (executive function, attention and working memory, memory, language, visuocognition)
- A value in the range of 1–2 standard deviations below the population mean should be used as a cut-off to define cognitive impairment
Level of consensus: 96.6%, strong consensus

*ACE* Addenbrooke’s cognitive examination; *ACE–R* Addenbrooke’s cognitive examination–revised version; *MoCA* Montreal cognitive assessment; *MDRS* Mattis dementia rating scale; *MMSE* Mini mental state examination; *PANDA* Parkinson neuropsychometric dementia assessment; *SCOPA–COG* Scales for outcomes in parkinson’s disease–cognition; *PDD* Parkinson’s disease dementia; *PD–MCI* Parkinson’s disease mild cognitive impairment

In contrast to the abbreviated Level I procedure, a comprehensive Level II neuropsychological assessment has shown better diagnostic accuracy for cognitive impairment in PwPD [25, 32]. The application of two tests per cognitive domain has indicated highest diagnostic accuracy for PD-MCI [33]. Normed neuropsychological tests across multiple cognitive domains have also consistently detected PD-related cognitive deficits compared to controls [34]. Recommendations for Level II diagnostic criteria for PD–MCI and PDD thus follow international consensus guidelines [16, 17].

#### Recommendations for the assessment of activities of daily living daily living activity impairment to support a diagnosis of Parkinson’s disease dementia

Significant ADL impairment associated with worsening cognition is a core criterion for PDD [16]. A differentiation can be made between basic ADL (BADL), which are necessary for self-maintenance (e.g., dressing, eating), and more complex instrumental ADL (IADL, e.g., managing finances) [35, 36]. Evaluating the potential of patient-, informant-, and investigator-rated ADL measures is still new. Diagnostic values have often not been confirmed in independent PD cohorts, but the assessments can differentiate between groups of PwPD with and without cognitive impairment (either PD–MCI or PDD) [37–41]. Therefore, applying scales has been limited to orienting screening procedures for further extensive interviews. To date, the Lawton and Brody informant Rating IADL Scale (cut-off < 19 points: sensitivity of 71%; specificity of 82%) [42], investigator-rated Unified Parkinson’s Disease Rating Sale II (UPDRS-II) (cut-off > 15 points: sensitivity of 67%; specificity of 78%) [42], Schwab and England Scale (cut-off < 75%: sensitivity of 71%; specificity of 77%) [42], Parkinson’s Disease Cognitive Functional Rating Scale (PD–CRFS, cut-off < 6 points: sensitivity of 83%; specificity of 83%) [43], and performance-based Pill Questionnaire (sensitivity of 89–91%, specificity of 89–91%) [42, 44] have been recommended as ADL screening tools for PwPD. The following German ADL assessments might also be considered as screening tools for ADL impairment to support a PDD diagnosis but have not been evaluated in international cohorts [43]:

- Nürnberger Alters Inventar Beobachtungsskala (NAB)–ADL (NAB–ADL, cut-off > 22 points: sensitivity of 81%; specificity of 80%)
- Nürnberger Alters Inventar Aktivitäten Skala (NAA)–ADL (NAA–ADL, cut-off > 34 points: sensitivity of 76%; specificity of 85%)
- Nurses’ Observation Scale for Geriatric Patients (NOSGER)–ADL (cut-off > 7 points: sensitivity of 67%; specificity of 86%)
- NOSGER–IADL (cut-off > 11 points: sensitivity of 62%; specificity of 67%)

**Table:** 

*Recommendations for instruments to assess activities of daily living impairment to support a diagnosis of Parkinson’s disease dementia (new)*
- For the exploration of ADL impairment, the following can be used: the informant-rated IADL questionnaire by Lawton and Brody, with a cut-off of <19 points; the UPDRS-II, with a cut-off of >15 points; the PD–CRFS with a cut-off of >6 points, the Schwab and England Scale, with a cut-off of <75%; the Pill Questionnaire
- The following scales might be used to assess ADL impairment: the NAB–ADL, with a cut-off of >22 points; the NAA–ADL with a cut-off of >34 points; the NOSGER–ADL, with a cut-off of >7 points; the NOSGER–IADL, with a cut-off of >11 points
If the test result is positive, further measures (e.g., medical history) should be utilized to verify whether ADL impairment is primarily caused by cognitive deterioration (expert opinion)
Level of consensus: 100%, strong consensus

*ADL* Activities of daily living; *IADL* Instrumental activities of daily living; *NAA-ADL* Nürnberger-Alters-Inventar Aktivitäten-Skala; *NAB-ADL* Nürnberger-Alters-Inventar Beobachtungsskala; *NOSGER-ADL* Nurses’ observation Scale for geriatric patients activities of daily living scale; *NOSGER-IADL* Nurses’ observation scale for geriatric patients instrumental activities of daily living scale; *PD-CRFS* Parkinson’s disease cognitive functional rating scale; *PDD* Parkinson’s disease dementia

### The non-pharmacological treatment of cognitive impairment in people with Parkinson’s disease

#### Cognitive interventions: general remarks

Non-pharmacological interventions, and especially cognitive interventions, have been considered promising therapeutic approaches to treat cognitive impairment in PwPD. Different forms of cognitive interventions can be distinguished: cognitive training, cognitive rehabilitation, and cognitive stimulation [45]. Cognitive training and rehabilitation appear to be particularly suitable for PD–MCI due to their level of difficulty and objectives. Cognitive stimulation, on the other hand, is more likely to be indicated for more advanced cognitive impairment or dementia. Cognitive training involves the use of standardized analog or digital tasks (e.g., with a laptop, tablet, or smartphone) that are directly aimed at training specific cognitive functions (e.g., executive function, attention, memory). It is often possible to adapt the difficulty level of the exercises to the individual’s cognitive level. Cognitive rehabilitation represents an individualized approach that is offered in practice as individual therapy. Individual everyday goals for cognitive rehabilitation are defined in collaboration with the patient and their relatives. Psychoeducation is often part of cognitive rehabilitation. Cognitive stimulation is usually conducted in a small group setting with a wide range of enjoyable activities to indirectly stimulate cognitive and social skills.

#### Cognitive training to treat Parkinson’s disease mild cognitive impairment

Studies evaluating treatment effects on PD–MCI in mixed cohorts of “non-demented” (with and without PD–MCI) or “cognitively impaired” (PD–MCI and PDD) PwPD were excluded from treatment recommendations, unless effects were separately reported for PD–MCI.

Among the seven RCTs comparing “classical” digital cognitive training with passive or active control groups, four showed that the experimental group was superior, indicating benefits to various cognitive outcomes like global cognition, executive function, attention, memory, visuocognition, and processing speed [46–49]. In two further studies, different cognitive and psychoeducational training (digital and non-digital) were compared, and they demonstrated positive effects on cognition in all training groups [50, 51]. Two RCTs evaluated the effects of digital training with gamification approaches, one of which used virtual reality training [52] and the other of which used cognitive training set up as a computer game [53]; both studies showed superiority in cognitive outcomes compared with analog cognitive training [52] and a passive control group [53]. Four RCTs that used analog cognitive training indicated superiority compared to active control groups in various cognitive outcomes [54–57].

However, the above-mentioned studies should be analyzed with caution, as most of them (1) had small sample sizes; (2) had different diagnostic criteria and used different test batteries (e.g., with or without using the MDS criteria for PD–MCI and PDD [15]); (3) lacked consideration of disease severity in the analysis of training effects; (4) had different outcomes, some of which were not clearly defined; (5) had heterogeneous training types (i.e., digital vs. analog training, individual vs. group training) that differed in duration and intensity. Therefore, recommendations for specific training characteristics were difficult to distinguish. Besides these limitations, current evidence has indicated the cognitive benefits of cognitive training in individuals with PD–MCI. This is consistent with the results of systematic reviews and meta-analyses evaluating the treatment effects of cognitive interventions in PwPD [58–60], which also include studies with PwPD without cognitive impairment or mixed cohorts (with and without PD–MCI). Cognitive training can thus be recommended for cognitive impairment. Furthermore, data have indicated that digital cognitive training can be particularly effective for cognitive impairment [52, 53]. However, analog training and training in both group and individual therapy settings have also shown positive effects [46–49]. Even if home-based training (especially digital) is available, the therapy should, if possible, be supervised in outpatient as well as inpatient settings. Training should also consider patients’ interests and circumstances (e.g., digital vs. analog training, group vs. individual training) to achieve a high level of therapy adherence. Multi-domain training (in which several cognitive domains are addressed, e.g., executive function, attention, and memory) appears to be appropriate since several cognitive domains are affected in most PD–MCI cases. However, training studies considering individual domains, especially executive function, have also shown positive effects and can therefore also be recommended. No statements can be made about transfer effects on non-trained cognitive and non-cognitive domains due to a lack of evidence, though. Regarding duration and frequency of the cognitive training, no clear recommendations can be drawn from the evidence. However, based on expert discussions, the authors recommend persistent cognitive training including several training sessions per week.

**Table:** 

*Recommendations for cognitive training to treat Parkinson’s disease mild cognitive impairment (new)*
Cognitive training can be offered
Level of consensus: 96.6%, strong consensus

#### Cognitive interventions to treat Parkinson’s disease dementia

To evaluate the effects of cognitive interventions to treat PDD, only studies in which individuals with PDD were analyzed as a separate study group were considered. Accordingly, the available studies that investigated and reported on mixed populations (e.g., individuals with PDD and Dementia with Lewy Bodies) were excluded.

Cognitive stimulation is recommended in the S3 guidelines for the diagnosis and treatment of dementia [6] (*should be offered*). However, no distinction is made between dementia types with different etiologies, so the specific effect on people with PDD is unclear. In the 2023 published Cochrane review on the evaluation of cognitive stimulation in (all-cause) dementia [61], 37 RCTs with a total of 2766 people with dementia were included. The authors concluded that cognitive stimulation shows small, short-term, positive effects on global cognition, communication, social interaction, quality of life, and psychological and behavioral symptoms in cases of mild to moderate dementia. However, again, dementia types were not differentiated. For people with PDD, one crossover pilot RCT investigated the effects of a cognitive stimulation program [62]. Despite the very small sample size (*N* = 12 in the experimental group, *N* = 6 in the control group receiving treatment as usual), there was a statistical trend indicating the superiority of the experimental group over the control group in global cognition, mood, and behavioral symptoms.

The German S3 guidelines for the diagnosis and treatment of dementia [6] also recommend reminiscence therapy for dementia with different etiologies. The Cochrane review published in 2018 [63] with a total of 22 RCTs involving 1972 people with dementia with different etiologies showed the positive effects of reminiscence therapy on cognition, quality of life, communication behavior, and mood. However, no specific evidence exists for people with PDD. Overall, a *might be used* recommendation was derived.

A Cochrane review on cognitive training in mild to moderate dementia with different etiologies published in 2019 [45] presented data indicating mild to moderate positive effects on global cognition and verbal fluency, but to date, no studies on PDD have been conducted. Furthermore, cognitive training and cognitive rehabilitation are not recommended in the S3 guidelines for people with dementia [6]. Therefore, no recommendations for cognitive training and cognitive rehabilitation for PDD were derived.

**Table:** 

*Recommendations for cognitive interventions to treat Parkinson’s disease dementia (new)*
Cognitive stimulation can be offered
Reminiscence therapy might be offered
Level of consensus: 96.8%, strong consensus

#### Physical training: general remarks

Endurance training and other forms of physical activity reduce the risk of cognitive impairment [64] and dementia in healthy older people [65]. Moreover, people with Alzheimer’s disease benefit from endurance exercise delivered in a dose-dependent manner, as it improves executive performance in particular [66]. Increasing evidence has demonstrated that physical training interventions may also improve cognition in PwPD [67]. Notably, a higher “lifetime physical training load” has been associated with better global cognition in PwPD [68].

#### Physical training to treat Parkinson’s disease mild cognitive impairment

Only physical training studies that defined cognition as a primary outcome were considered. Studies examining the effects of exergaming were excluded, as exergaming combines physical and cognitive training, so the specific effects of physical training could not be determined. In total, 23 studies were identified as relevant to the guidelines, only one of which focused exclusively on people with PD–MCI and not on mixed samples of PwPD with no cognitive impairment and PD–MCI.

The only RCT that used PD–MCI as an inclusion criterion (*N* = 50) showed the superiority of endurance training (6 times per week for 60 min over 4 weeks) for global cognition, attention, and working memory after 4 weeks compared to a passive control group. This superiority of the training group in global cognition was still present after 6 months [69].

Regarding trials with mixed samples of PwPD with and without cognitive impairment, one RCT (*N* = 76) indicated that endurance training is superior over an active control group (i.e., walking, stretching) in terms of its improvement of executive function over time, with both training types conducted 3 times per week for 60 min over 12 weeks [70]. Another small study (*N* = 9) with PwPD without cognitive impairment showed the superiority of treadmill training (3 times per week for 45 min over 3 weeks) in frontal functions compared to a passive control group [71]. In a non-randomized study with 11 PwPD participating in endurance training (3 times per week for 60 min over 6 months), the training group was again superior to a passive control group in executive function [72]. A systematic review of RCTs also showed an advantage of physical activity over passive control groups in the areas of global cognition, cognitive flexibility, attention, and cognitive processing speed [73].

For a detailed description of the recommendation, one systematic review [73], two RCTs [69, 70], and a non-randomized study evaluating physical exercise training [71] were used, the latter because the study defined cognitive variables as its primary outcome. As the above mentioned studies varied in time per session (45–60 min), frequency of session per week (3–6 times) and the total number of training weeks (3–24 weeks), the recommendation for the duration and frequency of physical exercise training was based on an expert consensus considering the current state of knowledge [69–73] as well as practical issues to conduct physical training in the clinical care (e.g. PwPD with a long journey might not be able to conduct 3 sessions per week). The optimal training intensity and frequency for PD-MCI have not been investigated yet, but should be the focus of future studies. Additionally, no differentiation regarding the type of endurance training could be made.

**Table:** 

*Recommendations for physical interventions to treat Parkinson’s disease mild cognitive impairment (new)*
Physical (aerobic) training should be conducted 2–3 times per week for 45–60 min to treat PD–MCI
Level of consensus: 96.8%, strong consensus

#### Physical training to treat Parkinson’s disease dementia

Only three studies with very small sample sizes comprising people with PDD diagnosed according to established criteria (*n* < 5) were identified [74–76]. Given the lack of sufficient data, the expert team decided that no recommendation could be derived for physical training to treat PDD.

**Table:** 

*Recommendations for physical interventions to treat Parkinson’s disease dementia (new)*
Data are insufficient to recommend physical training to treat cognitive symptoms in PDD
Level of consensus: 96.7%, strong consensus

#### Food supplements and diet to treat Parkinson’s disease mild cognitive impairment and Parkinson’s disease dementia

Evidence for food supplements, herbal therapeutics, and specific diet approaches as treatment options for PD–MCI and PDD is currently mainly limited to narrative review articles and expert opinions, and no double-blinded placebo-controlled RCT has been published. Ketogenic [77] and Mediterranean diets [78], creatinine, and coenzyme Q10 [79] have demonstrated small improvements in cognitive function in people with PD–MCI, whereas the results of caffeine have been mixed [80, 81]. One study evaluating the effects of a combination of donepezil, dl-3*n*-butylphthalide oxiracetam, and Ginkgo biloba in people with PDD showed slight improvements in their global cognitive state [82]. However, data were regarded as insufficient to derive any recommendation therefrom.

**Table:** 

*Recommendations for food supplements and herbal therapeutics to treat Parkinson’s disease mild cognitive impairment or Parkinson’s disease dementia (new)*
Food supplements and herbal therapeutics should not be used to treat cognitive deficits in PD–MCI
Level of consensus: 96.6%, strong consensus
Food supplements and herbal therapeutics should not be used to treat cognitive deficits in PDD
Level of consensus: 96.6%, strong consensus

### The pharmacological treatment of cognitive impairment in people with Parkinson’s disease

#### General remarks

Reduced cholinergic activity has been demonstrated in people with PDD [83, 84], but altered cholinergic innervation is already present in the early stage of PD [85–87]. Deficits in executive function, attention, and memory have been correlated with cholinergic activity assessed via cholinergic [18F] fluoroethoxybenzovesamicol positron emission tomography in PwPD [85]. Memantine is a moderate affinity uncompetitive antagonist of glutamate *N*-methyl-d-aspartate receptors and is effective for treating cognitive dysfunction in people with Alzheimer’s disease [88]. Therefore, the efficacy of AChEIs and memantine for cognition has not only been evaluated in PDD but also in PD–MCI.

#### Pharmacological interventions to treat Parkinson’s disease mild cognitive impairment

Studies evaluating the effect of AChEIs on PD–MCI are limited. A trend in favor of rivastigmine treatment (maximum dose of 9.5 mg per 24 h) in the clinical global impression of change as a primary outcome in a 24-week, randomized, double-blinded, placebo-controlled trial [89] but not in cognitive scales, behavior, quality of life, and ADL was shown. Open-label treatment with donepezil (5–10 mg) for 48 weeks in 80 people with PD–MCI showed no change in their global cognitive state [90]. However, treatment with donepezil had a modulatory effect on the electroencephalography of people with PD–MCI. In a mixed group of 145 people with no cognitive impairment and PD–MCI, the efficacy of donepezil (5 mg per day) to improve verbal memory as a secondary outcome but not their global cognitive state was reported in a double-blinded RCT [91, 92]. No study on treatment with galantamine in people with PD–MCI could be identified.

Only one RCT with a crossover design (6-week treatment period) analyzed the effect of memantine (20 mg per day) on 10 people with PD–MCI [93]. It was found that psychomotor speed and working memory worsen under treatment with memantine, but other neuropsychological tests showed no treatment-induced change.

**Table:** 

*Recommendations for acetylcholinesterase inhibitors to treat Parkinson’s disease mild cognitive impairment (new)*
Rivastigmine, donepezil, and galantamine should not be used to treat PD–MCI
Level of consensus: 89.7%, consensus
*Recommendations for memantine to treat Parkinson’s disease mild cognitive impairment (new)*
Memantine should not be used to treat PD–MCI
Level of consensus: 96.7%, strong consensus

#### Pharmacological treatments for Parkinson’s disease dementia

Evidence for AChEI treatment has been updated. Data from a meta-analysis confirmed the positive treatment effects of rivastigmine and donepezil on PDD patients’ global cognitive state [94], memory, and speech [95]. However, there is stronger evidence for rivastigmine in meta-analyses. First, meta-analyses did not consistently report positive effects of donepezil on cognitive tests, and positive effects of donepezil on ADL functioning has not been demonstrated yet [96, 97]. In this guideline, we rate positive changes in cognition (“pathophysiological validity”) and ADL functions (“ecological validity”) higher than a change in the “Clinical Global Impression of Change”, as this is dichotomized in meta-analyses (“improvement” versus “stable or worsening”) and thus loses some of its statistical significance. Both, changes in cognition (better effectiveness) and positive ADL functions favors rivastigmine [96, 97]. Improvements in neuropsychiatric symptoms have been verified with rivastigmine but not donepezil [94, 95, 98]. Otherwise, it has been shown that treatment with donepezil compared with a placebo leads to improvements in ADL [96]. The occurrence of side effects worsening PD symptoms and tremors has been reported with rivastigmine use but is less prominent with donepezil use [94, 95]. No effect for galantamine treatment has been identified [98].

Two meta-analyses [88, 99] reported only small improvements in cognitive function and global clinical impressions for memantine in people with PDD, but treatment effects were considerably higher in mixed cohorts with PDD and Lewy body dementia.

**Table:** 

*Recommendations for acetylcholinesterase inhibitors in the treatment of Parkinson’s disease dementia (updated*)
Rivastigmine should be used
Donepezil might be used (off-label use)
Galantamine cannot be used
Level of consensus: 96.6%, strong consensus
*Recommendations for memantine in the treatment of Parkinson’s disease dementia (new)*
Memantine should not be used
Level of consensus: 96.6%, strong consensus

Table 1 gives an overview of the recommendations for the non-pharmacological and pharmacological treatment of PD–MCI and PDD.

**Table 1:** Overview of recommendations for pharmacological and non-pharmacological treatment of PD-MCI and PDD

	Non-pharmacological treatment			Pharmacological treatment			
	Cognitive intervention	Physical intervention	Nutrition	Memantine	Rivastigmine	Donepezil	Galantamine
PD-MCI	++ Cognitive training	+++ 3×/week aerobic training	–	–	–	–	–
PDD	++: Cognitive stimulation; + remisniscence therapy	–	–	–	+++	+ Off-label use	–

+++ should be offered; ++ can be offered; + might be offered; – no recommendation

## Affective disorders

### Definition, epidemiology, and clinical presentation

Affective disorders are highly debilitating and commonly occurring in PwPD. For example, a meta-analysis of 30 studies including *N* = 7142 PwPD with a disease duration of more than 3 years found a prevalence of depressive disorders of 47.2%, apathy of 45.5%, and anxiety disorders of 42.9% [1].

However, affective disorders in PwPD are often underdiagnosed and, therefore, untreated, which is likely to have a significant negative impact on their quality of life and overall prognosis [100–104]. For example, in a survey of diagnostic accuracy at the University of Miami, Florida, USA, neurologists overlooked every second case of depression in PwPD with outpatient contact [104]. Further studies have shown that only a quarter of PwPD with depressive disorders receive antidepressant treatment [105, 106].

#### Depression, anhedonia, and mood swings

According to the draft version of the *International Statistical Classification of Diseases and Related Health Problems* revision 11 (*ICD-11*) [107], depressive disorders are characterized by a depressed mood (e.g., feeling sad, irritable, empty) or joylessness (i.e., anhedonia), accompanied by other cognitive, behavioral, or neurovegetative symptoms that significantly impair the person’s ability to function. Studies with PwPD have indicated that there is a close negative correlation between patients’ quality of life and the extent of their depressive symptoms, while there is no or a weaker correlation with motor impairments [108, 109]. In a meta-analysis on the prevalence of depressive disorders in PwPD, Cong et al. (2022) [110] found a prevalence of 38% based on 129 studies with *N* = 38,304 PwPD. It was also found that depressive disorders are associated with longer disease duration, higher disease severity, and more motor symptoms (especially bradykinesia) and non-motor symptoms (apathy, anxiety disorders, fatigue). Anhedonia, or the inability to feel pleasure, can be a symptom of both depressive and apathetic syndromes [111], and its prevalence has been described to be between 10 and 40% [112]. Studies have indicated that 40–50% of PwPD report motor fluctuations, while much less is known about non-motor fluctuations, which often occur in the domain of emotion and affect. A systematic review indicated that 35% of PwPD experience feelings of anxiety, while 35% also experience symptoms of depression. Panic attacks are also common among PwPD [113].

#### Apathy

Apathy is defined as a lack of motivation characterized by reduced goal-oriented thinking and action, as well as reduced emotional expression. It is, therefore, a drive disorder that can have a negative impact on the motivation for self-care and is often a major burden for relatives [101]. Meta-analytic data have indicated a prevalence of 40% for apathetic symptoms in PwPD, with the occurrence of apathy being associated with older age, cognitive impairment, the presence of depressive disorders, and more advanced PD symptoms [101].

#### Anxiety disorders and fear of progression

According to the *ICD-11* [107], anxiety disorders are defined as mental disorders characterized by excessive fear and anxiety and associated behavioral disturbances, with symptoms severe enough to cause significant distress or impairment in personal, familial, social, educational, occupational, or other important areas of functioning. Various diagnoses can be considered anxiety disorder diagnoses, including generalized anxiety disorder, panic disorder, agoraphobia, specific phobias, or social anxiety disorder. In a systematic review with a meta-analysis based on 45 studies with *N* = 2399 PwPD, a prevalence of 31% for anxiety disorders in general was identified, with generalized anxiety disorder being the most common type of anxiety disorder at 14% [100]. Furthermore, the point prevalence for social anxiety disorder was 14%, 13% for unspecified anxiety, 13% for specific phobias, and 7% for panic disorder. During the course of chronic diseases, fear of progression has also been described. Fear of progression is a real fear that arises from the experience of a serious, potentially life-threatening, or disabling disease and its diagnosis and treatment [114]. In terms of differential diagnoses, fear of progression must be clearly distinguished from anxiety disorders. In a study that compared chronic diseases in terms of the extent of their associated fear of progression, PD showed the second-highest values [114]. In a cross-sectional study that Folkerts et al. [115] conducted with *N* = 120 PwPD, moderate levels of fear of progression were found in 63% of respondents, while 18% showed dysfunctional levels of fear of progression.

#### Fatigue

Fatigue in the context of PD is defined as an (almost) daily feeling of “a markedly reduced energy level or increased perception of exertion out of proportion to the activities attempted or the general level of activity” [116]. A distinction can be made between physical fatigue (i.e., the feeling of physical exhaustion or a lack of physical energy) and mental fatigue (i.e., the cognitive effects that occur during and after prolonged periods of sustained mental exertion) [117]. The reported prevalence of fatigue in PwPD has varied widely, ranging from 33 to 70%. It has been found that fatigue is more common in later stages of PD [118]; however, it can also manifest in the premotor stages of PD [119]. Fatigue impairs ADL and significantly limits the social and occupational participation and quality of life of PwPD [120].

### The diagnosis of affective disorders in people with Parkinson’s disease

#### General remarks: challenges in diagnosing affective disorders in people with Parkinson’s disease

The diagnosis of affective disorders in PwPD is challenging, particularly because the signs and symptoms of affective disorders can overlap with motor and other non-motor symptoms. In addition, the diagnostic criteria for depressive disorders that have been established for people without PD, for example, may not be readily applicable to PwPD, as the spectrum of depressive symptoms is not entirely the same [121]. Depressive symptoms in PwPD often include dysphoria, pessimism, anxiety and somatic symptoms, and less frequently, feelings of guilt, failure, self-blame, or suicidality [122–124]. Moreover, feelings of emptiness and hopelessness, reduced responsiveness to emotional stimuli, or loss of the ability to enjoy and feel pleasure (anhedonia) have been described in PwPD [125]. The clinical diagnosis of depression in PwPD usually refers to the presence of such symptoms and not to morning dysphoria, weight loss, or too much or too little sleep [121].

A further challenge for the diagnosis of depressive disorders in PwPD often arises from concomitant dementia. On one hand, possible symptoms of a depressive disorder can also be symptoms of dementia (e.g., anhedonia, irritability [121]); on the other hand, cognitive disorders can also be caused by depression. In advanced PD, an overlap between depression and dementia is common and represents a challenge for differential diagnosis.

Apathy can occur in PwPD in isolation, as an expression of depression, as a fluctuation in mood, or in the context of motor and cognitive symptoms. Specifically, the clinical differentiation of apathy from the affective symptoms of depression and executive dysfunctions, such as in planning and organizing, is challenging [126].

Although fatigue in PwPD is often associated with daytime sleepiness, depression, and apathy as a comorbidity, it can and should be separated from these as an independent phenomenon by, among other things, obtaining a careful medical histor﻿y﻿ [127, 128].

#### Questions to be asked when obtaining a patient’s medical history to assess affective disorders

Obtaining a careful medical history plays a crucial role in detecting affective disorders during routine clinical practice. On one hand, there is limited time and personnel resources for the use of (semi-)structured clinical interviews or standardized scales as self-evaluation or other-evaluation with which affective disorders can be recorded or their severity evaluated. On the other hand, scales should not be used as a sole instrument for diagnosing affective disorders. Notably, depression cannot be diagnosed solely based on a score, as high depression scores can occur with somatic symptoms despite the absence of core symptoms of depression (e.g., sadness, loss of interest, anhedonia), and low scores can occur despite the presence of severe depressive symptoms if somatic or vegetative problems are absent. All depression scales contain items on symptoms that can be associated with depression, in addition to apathy, cognitive impairment, and parkinsonism. This applies in particular to the Hamilton Depression Rating Scale (HAM-D) and, to a lesser extent, the Montgomery–Asberg Depression Rating Scale (MADRS) and Beck Depression Inventory (BDI) [128].

Furthermore, scales generally record patients’ subjective state of health over the preceding 1 or 2 weeks. In addition, they do not consider the objective clinical condition of patients with fluctuating motor function. This means that PwPD may perceive their own condition differently when they are in an off-status than when they are in an on-status [129]. Off-periods can also be associated with fluctuating non-motor symptoms, depression, anxiety, and delusions [121, 129, 130].

When obtaining a medical history, the question arises as to which questions are suitable for the sensitive and specific assessment of affective disorders. Based on the above-mentioned evidence and considerations, the authors suggest the following questions for the assessment of affective disorders, per expert consensus. The number and selection of questions are applicable to, sensible in, and practicable to everyday clinical practice. When obtaining a medical history, patients should be in a clinical on-status, which should be documented using the motor examination of the UPDRS-III if necessary. In a recognizable clinical off-status, medication should be administered first, or the result should be documented as “conditional” and checked when the patient is in an on-status. An external medical history can provide valuable information for supplementary diagnostics. This applies in particular to the presence of cognitive disorders in PwPD.

**Table:** 

*Recommendations for questions to be asked when obtaining a medical history to assess affective disorders (new)*
*Affective disorders—general: The two questions from the “two-question test” should be asked*
Have you often felt down, sad, depressed, or hopeless in the last month?
In the past month, have you had significantly less pleasure and joy in doing things that you normally enjoy?
*Depression*
Have you ever suffered from a depressive disorder, and have you ever received antidepressant treatment?
Do you sleep well or sleep through the night?
How is your appetite?
Do you often feel an inner restlessness?
Are you still able to enjoy things?
Do you have difficulty concentrating or staying alert?
Does your mood change when your medication wears off or stops working?
*Apathy and mood swings*
Are you still interested in activities, things, or people?
Do you sleep a lot during the day?
Do you do things with friends or family?
Does your drive and motivation change when your medication wears off or stops working?
*Anxiety disorders and mood swings*
Are you often anxious?
Do you often feel an inner restlessness or tenseness?
Are you prone to panic attacks?
Are you afraid of being around people?
Do you experience feelings of anxiety when the effect of your medication decreases or wears off?
*Fear of progression*
Are you afraid of the future course of your disease?
Are you pessimistic, despondent, or hopeless about the future when you think about the further course of your Parkinson’s disease?
*Fatigue and mood swings*
Do you often feel tired or exhausted, despite getting enough sleep?
Do you often lack the energy to do things?
Do you sleep a lot during the day?
Do you have difficulty concentrating or staying alert?
Do you feel exhausted when your medication wears off or fails to work?
Level of consensus: 93.4%, strong consensus

#### Scales and questionnaires

In addition to data derived from systematic reviews and previously published guidelines, the recommendations of the MDS were considered. Besides relevant information that is gathered from a self- and externally obtained medical history, various general non-motor symptom scales or relevant subscales and the generic Neuropsychiatric Inventory (NPI) can be considered. However, the assessment of the affective symptoms in all these scales is based on individual or only a few items, and in some cases, no separate scores for the specific affective disorders are available. The gold standard for diagnosing affective disorders in PwPD according to the MDS is to conduct a (semi-)structured interview based on the criteria of the *Diagnostic and Statistical Manual of Mental Disorders* fifth edition (*DSM-5*, i.e., with the Structured Clinical Interview for *DSM-5* Disorders [SCID-5]). However, this standard is rarely implemented in clinical settings. If there are signs of affective disorders, several scales are available that assess the specific types of these disorders, including self-assessment questionnaires and external assessment scales. To differentiate the severity of depressive disorders, only specific depression scales seem valuable. Due to the clinical relevance of this symptom, these guidelines also suggest a questionnaire for the assessment of fear of progression. The idea of a regular screening package to monitor possible affective disorders in PwPD was raised, e.g., by sending a questionnaire package by post in advance of a routine neurological examination.

**Table:** 

*Recommendations for scales and questionnaires to assess affective disorders (new)*
The following scales and questionnaires are recommended
*Initial diagnosis of affective disorders: General scales*
Scales for the assessment of non-motor symptoms (here, specific items or subscales for affective disorders) can be used
- Movement Disorders Society Unified Parkinson’s Disease Rating Scale, Part 1 (MDS-UPDRS-I)
- Movement Disorders Society Non-Motor Rating Scale (MDS-NMS)
- Non-Motor Symptom Scale (NMSS)
- Non-Motor Symptom Questionnaire (NMSQ)
*Initial diagnosis of affective disorders: Specific scale*
The corresponding items of the following instrument might be used:
- Neuropsychiatric Inventory (NPI)
*Specific scales*
For the specific diagnosis of affective disorders, specific scales validated for PwPD should be used. The following scales are suitable for this purpose, are frequently used in everyday clinical practice, and are recommended by the authors
*Depression*
- Beck Depression Inventory (BDI)-II
- Geriatric Depression Scale (GDS-15)
- Two-question test
*Anhedonia*
- Snaith–Hamilton Pleasure Scale (SHAPS)
*Apathy*
- Apathy Evaluation Scale (AES)
*Anxiety*
- Hospital Anxiety and Depression Scale (HADS)
*Fatigue*
- Fatigue Severity Scale (FSS)
*Fear of progression* (might be used)
- Short form of the Fear of Progression Questionnaire (FoP-Q)
*Depression: Assessment of the degree of severity* (might be used)
- BDI-II
Level of consensus: 100%, strong consensus

### The non-pharmacological treatment of affective disorders in people with Parkinson’s disease

#### General remarks

In this chapter, the authors considered only interventions that, according to current German routine clinical practice, physicians may prescribe for PwPD. These include psychotherapy, physical interventions, and occupational therapy. As data on occupational therapy were insufficient, no recommendations could be derived therefrom.

#### Depression

Regarding psychotherapy, recommendations were based on two meta-analyses that analyzed the effects of CBT [131, 132] and one meta-analysis that considered the effects of both CBT and psychodynamic approaches [133] in PwPD. All these analyses showed significant effects of psychotherapy on depression compared to control groups. The effects of psychotherapy might even have been larger than the effects of antidepressants [134]. Notably, a subgroup analysis of one meta-analysis [133] found that treatments of more than 6 weeks are more effective than shorter treatment periods. A subgroup analysis of another meta-analysis confirmed that longer interventions (here, 8 or more weeks) have a greater effect than shorter intervention durations [132]. This latter study also indicated that individual interventions are significantly more effective than group interventions in treating depression in PwPD, while the effects of online and offline interventions do not differ.

Two overarching meta-analyses that included studies on different physical interventions in PwPD [135, 136] and several meta-analyses on specific physical interventions (e.g., dance, resistance training) [137–142] demonstrated convincing evidence for their effectiveness on depressive symptoms in PwPD. Furthermore, the German guidelines for unipolar depression (2022) [7] recommend motivating patients with a depressive disorder to engage in physical activity, ideally within a group; they should also be motivated to participate in structured and supervised physical training, and they should be supported in its implementation. Notably, there should be no contraindication for the specific type of physical exercise. and in PwPD, the risk of falling should be given special consideration when choosing and intensifying physical activity.

**Table:** 

*Recommendations for non-pharmacological interventions to treat depressive symptoms (new*)
Cognitive behavioral therapy should be offered
Physical interventions should be offered
Level of consensus: 100%, strong consensus

#### Apathy

No meta-analysis or RCT on psychotherapeutic interventions for the treatment of apathy in PwPD was identified. Furthermore, apathy has rarely been defined as an outcome in physical intervention studies. Two meta-analyses [138, 139] that both included the same two RCTs (*N* = 62 PwPD) that defined apathy as an outcome found no significant effect of dance therapy on apathy scores compared to active control groups. However, an RCT with *N* = 20 PwPD showed a positive effect of Nordic walking on apathy symptoms compared to a passive control group [143]. An RCT that investigated the effectiveness of aerobic training compared to a stretching program among *N* = 35 PwPD was unable to show the superiority of either training program [144]. The comparison of a physical intervention held in a group format and individual format without supervision among *N* = 30 PwPD was also unable to show the superiority of one type of training in relation to apathy symptoms [145].

Based on the one RCT that provided evidence for a possible effect of physical interventions for the treatment of apathetic symptoms in PwPD, the following recommendation was proposed.

**Table:** 

*Recommendations for non-pharmacological interventions to treat apathy (new)*
Physical interventions might be offered
Level of consensus: 100%, strong consensus

#### Anxiety

Two recent meta-analyses [131, 132] examining the effects of psychotherapy on anxiety were identified; both considered only CBT. Zhang et al. [131], who included five RCTs and one non-RCT, with *N* = 163 PwPD in their meta-analysis found a significant moderate effect of CBT offered in different formats (individual vs. group therapy, analog vs. telephonic therapy) compared to the active and passive control groups. In the meta-analysis published in 2021 by Luo et al. [132], ten studies with *N* = 383 PwPD were included in the comparison of CBT and control groups, showing a significant and large effect in favor of CBT. Subgroup analyses also demonstrated that interventions over a period of at least 10 weeks have the greatest effect on reducing anxiety. There were no differences between the effects of analog and digital formats. However, individual therapy proved to be superior to group therapy. Notably, in the German S3 guidelines on the treatment of anxiety disorders [8], CBT is given the highest recommendation for the treatment of generalized anxiety disorder, panic disorder or agoraphobia, specific phobias, and social anxiety disorder. No meta-analysis or RCT examining the effects of psychotherapy on fear of progression was identified.

Regarding physical interventions, a recent large-scale network meta-analysis [136] that considered 250 studies with *N* = 13,011 PwPD was identified. Thirteen of these studies with *N* = 757 PwPD included anxiety symptoms as an outcome. The network meta-analysis only found yoga to be significantly superior to the active and passive control groups. However, this result was based on only one yoga study. According to the authors of another recent meta-analysis [146] that focused specifically on the effects of physical interventions on anxiety symptoms in PwPD, currently available data were insufficient to derive any recommendations.

Based on the given evidence, the following recommendation was formulated.

**Table:** 

*Recommendations for non-pharmacological interventions to treat anxiety (new)*
Cognitive behavioral therapy should be offered
Level of consensus: 100%, strong consensus

As there is an overlap between anxiety disorders and fear of progression, the use of CBT for severe fear of progression was also discussed in the expert meetings. Here, RCTs with other target groups (e.g., people with cancer) that showed positive effects of CBT on fear of progression were also considered (e.g., [147]). The recommendation was formulated as follows.

**Table:** 

*Recommendations for non-pharmacological interventions to treat fear of progression (new)*
Cognitive behaivoral therapy might be offered
Level of consensus: 100%, strong consensus

#### Fatigue

Data on the effects of psychotherapeutic approaches for fatigue management have not been convincing. In their meta-analysis, Luo et al. [132] included two studies on CBT compared to active control groups with *N* = 32 PwPD and were unable to identify a significant effect on fatigue symptoms. Jiang et al. [148] included only one RCT with *N* = 12 PwPD in their meta-analysis, which investigated a combination of CBT and light therapy compared to a placebo group, but found no positive effects on fatigue symptoms. A recent RCT that Bogosian et al. [149] conducted also did not demonstrate any significant effects of a mindfulness-based intervention on the fatigue symptoms of PwPD compared to a passive control group. Therefore, no recommendation for psychotherapy as a treatment for fatigue was given.

Only two RCTs with *N* = 57 PwPD participating in physical interventions could be included in the 2015 Cochrane review [150], whereby no positive effect on fatigue symptoms could be determined. Two meta-analyses on specific physical intervention approaches (i.e., mind–body interventions and dance therapy) only included three ([138]; *N* = 74 PwPD) and two ([138]; *N* = 58 PwPD) RCTs, respectively, and could not show the superiority of physical interventions over control groups. A recent meta-analysis [148] with four RCTs and *N* = 100 PwPD participating in different physical intervention approaches showed a trend for the superiority of the intervention over control groups. Another recent meta-analysis [151] was able to draw on 30 RCTs on different drug and non-drug treatment approaches. Eight studies with *N* = 324 PwPD could be considered for meta-analysis on physical interventions in comparison with control groups. There was shown a significant but small effect of physical interventions on fatigue symptoms in PwPD compared to the active and passive control groups. No clear recommendations regarding duration, frequency and type of intervention can be drawn from the existing evidence. Based on these considerations, the following recommendation was given.

**Table:** 

*Recommendations for non-pharmacological interventions to treat fatigue (new)*
Physical interventions should be offered
Level of consensus: 100%, strong consensus

An overview of the recommendations for non-pharmacological intervention approaches to treat affective disorders in PwPD is given in Table 2.

**Table 2:** Overview of recommendations for non-pharmacological treatment of affective disorders in PwPD

	Cognitive behavioural therapy	Physical intervention
Depression	+++	+++
Apathy	–	+
Anxiety	+++	–
Fear of progression	+	–
Fatigue	–	+++

+++ should be offered; + might be offered; – no recommendation

### The pharmacological treatment of affective disorders in people with Parkinson’s disease

#### Dopaminergic therapy in the treatment of affective disorders

The optimization of dopaminergic replacement therapy is an essential factor in the treatment of non-motor fluctuating and non-fluctuating, affective symptoms in PwPD [121, 152].

#### Depression

An RCT (*n* = 287 PwPD were randomized 1:1 to a treatment with pramipexole or a placebo) showed a significant improvement in BDI scores at 12 weeks in the pramipexole group compared with the placebo group, with an acceptable safety profile [153]. Three meta-analyses showed an antidepressant effect of pramipexole in PwPD [154–156]. In an RCT involving 44 PwPD, pramipexole and citalopram led to a comparable reduction in BDI scores after 8 weeks [157]. An RCT testing the antidepressant effect of rotigotine in 80 PwPD (randomized 1:1 to a placebo group) did not show superiority of the antidepressant [158]. Two meta-analyses, including eight and 10 RCTs, respectively, showed a positive effect of rotigotine on depressive symptoms in PwPD [159, 160]. Overall, there has been limited evidence for the effects of piribedil on depressive symptoms in PwPD.

#### Anhedonia

No RCT with anhedonia as a primary outcome could be found. Anhedonia is thought to reflect a hypodopaminergic state in PwPD [161]. A single dose of l-dopa improves anhedonia in patients with advanced PD [152]. Observational studies and secondary analyses have also reported beneficial effects of rotigotine, pramipexole, and piribedil on anhedonia symptoms [162–164].

#### Mood swings

No RCT with mood swings as a primary outcome could be found. As with motor fluctuations, experts recommended stable plasma l-dopa levels and non-ergot dopamine agonists [165].

#### Apathy

Apathy has been shown to improve with l-dopa therapy [166]. Twenty-two PwPD were treated with pramipexole, ropinirole, or l-dopa, and pramipexole was shown to have a better effect on symptoms of apathy than ropinirole and l-dopa [167]. A meta-analysis and several case reports and observational studies showed a positive effect of pramipexole and rotigotine on apathy symptoms [162–164, 168]. A placebo-controlled RCT showed that piribedil can be used successfully for the treatment of symptoms of apathy after deep brain stimulation of the subthalamic nucleus [168]. An RCT for the treatment of apathy in de novo PD patients did not show superiority of ropinirole [169].

#### Anxiety disorders

Studies have indicated that anxiety symptoms associated with mood swings can be successfully treated with l-dopa and non-ergot dopamine agonists. This has been shown in case reports, observational studies, and two meta-analyses [170–172]. Trials defining anxiety as a non-fluctuating symptom as their primary outcome are lacking, though.

#### Fatigue

In the ELLDOPA study, 361 de novo PwPD received a placebo or l-dopa at various doses for 40 weeks. The l-dopa groups showed significantly less progression in fatigue during the study [173]. This finding is consistent with the observation that l-dopa reduces fatigue [174]. Two studies (including one RCT) reported an improvement in fatigue with rotigotine [162, 175]. Two meta-analyses, including 10 and 8 studies, respectively, with a total of 1800 PwPD reported a positive treatment effect of rotigotine on fatigue symptoms [159, 160]. Pramipexole’s effect on fatigue symptoms has been less thoroughly studied, but available studies have provided evidence for both improvements and worsening [176, 177].

**Table:** 

*Recommendations for dopaminergic medication to treat affective disorders in people with Parkinson’s disease (new)*
*Depressive disorders*
An optimal dopaminergic medication should be used to treat depressive disorders. Pramipexole should be used if dopamine agonist therapy is individually feasible. The dopamine agonist rotigotine might be used as a secondary treatment
*Anhedonia*
The optimal dopaminergic medication of levodopa and/or rotigotine, pramipexole, or piribedil should be used to treat anhedonia
*Mood swings*
An optimal dopaminergic medication should be used for the treatment of mood swings. Non-ergot dopamine agonists might be used. No dopamine agonist has been shown to be superior, though
*Apathy*
An optimal dopaminergic medication should be used to treat apathy. In addition, the dopamine agonists pramipexole, rotigotine, or piribedil might be used if dopamine agonist therapy is individually feasible
*Anxiety disorders*
For anxiety disorders with mood swings, the optimal dopaminergic medication should be used, and a non-ergot dopamine agonist should be used if dopamine agonist therapy is individually feasible
Dopaminergic therapy cannot be used to treat persistent anxiety without mood swings, though
*Fatigue*
An optimal dopaminergic medication should be used to treat fatigue. In addition, rotigotine might be used if dopamine agonist therapy is individually feasible
Level of consensus: 100%, strong consensus

An overview of the recommendations for the dopaminergic treatment of affective disorders in PwPD is given in Table 3.

**Table 3:** Overview of recommendations for dopaminergic treatment of affective disorders in PwPD

	Individually optimized treatment with levolopa	Non-ergot dopamine agonists (if individually feasible, monotherapy or add on with levodopa)			
	Levodopa	Pramipexole	Rotigotine	Piribedil	Ropinerole
Depression	+++	+++	+	–	–
Anhedonia	+++	+	+	+	–
Apathy	+++	+	+	+	–
Anxiety with affective fluctuations	+++	+	+	+	+
Anxiety without affective fluctuations	–	–	–	–	–
Fatigue	+	–	+	–	–

+++ should be offered; + might be offered; – no recommendation

#### Non-dopaminergic therapy in the treatment of affective disorders: General remarks

In addition to the dopaminergic system, other neurotransmitter systems are also involved in the evolution of affective disorders in PwPD. Serotonergic degeneration has already been found to play a prominent role in de novo PwPD [178, 179]. The role of the glutamatergic system as a modulator of emotions has also been discussed [180]. The national guidelines for unipolar depression [7] point out that patients with severe depression (“major depression”) in particular benefit from antidepressant drug therapy, while this effect is not so clear in people with mild depression (“minor depression”) or dysthymic disorder, and it is unclear whether this finding can also be applied to PwPD suffering from depression. In addition, most PwPD with signs and symptoms of depression do not fulfill the criteria for major depression [108].

#### Depressive and anxiety disorders

The previous guidelines for PD, published in 2016 [181], recommended tricyclic antidepressants (TCAs), selective serotonin reuptake inhibitors (SSRIs), venlafaxine, and omega-3 fatty acids for the treatment of major depression in PwPD. As a basis, a meta-analysis showed no difference in the treatment of depression in PwPD between SSRI and a placebo and TCA [182]. A systematic review [183] reported that nortriptyline (two RCTs [184, 185]) and desipramine (one RCT [186]) are probably effective for the treatment of depressive disorders in PwPD. One study showed that nortriptyline, but not paroxetine, is effective compared with a placebo in improving depressive symptoms in PwPD in an 8-week RCT [184]. In the 16-week blinded extension phase, relapse was significantly more frequent with the placebo than with nortriptyline and paroxetine [186, 187]﻿. In the RCT, the antidepressant effect of desipramine occurred earlier (after 14 days) than that of citalopram (after 30 days) [186]. Another RCT without a placebo arm showed that low-dose amitriptyline and sertraline have comparable antidepressant effects [188]. A 4-week RCT with 23 PwPD showed that treatment with 50 mg of 5-hydroxytryptophan (5-HTP), which is an intermediate metabolite of l-tryptophan in the production of serotonin, results in significant improvements in depressive symptoms (using the HAM-D) compared with a placebo. There was, however no effect of 5-HTP on symptoms of apathy [189]. A 12-week RCT with omega-3 fatty acids showed a significant reduction in depression scores compared with a placebo [190]. Supplementary Table 2 shows an overview of prospective controlled studies on treatment with non-dopaminergic substances in PwPD with depression. Notably, the study period has been short in most studies, and the number of cases in the study populations analyzed has been small.

Antidepressants with anticholinergic properties are associated with an increased risk of deterioration in cognitive function, triggering hallucinations or psychosis, increased constipation, and an increased cardiovascular risk, particularly in elderly and cognitively impaired PwPD, and should only be used with caution [121]. The noradrenaline reuptake inhibitor desipramine demonstrates both efficiency as an antidepressant [186] and a smaller anticholinergic and antihistaminic effect among TCAs. Desipramine is not commercially available in the EU but can be imported (as norpramine) from international pharmacies in drug dosages of 10, 25, 50, 100, or 150 mg. Nortriptyline, the TCA with the best data among PwPD, acts mainly as a serotonin and noradrenaline reuptake inhibitor (SNRI), in addition to its sedative effect due to its additional antagonization of H1 and 5-HT2A receptors [191]. However, nortriptyline has a high affinity for antagonizing muscarinic acetylcholine receptors and, therefore, has a strong anticholinergic effect [191]. In addition, there is a potential cardiotoxic effect due to the sodium or calcium channel blocking action [192] of nortriptyline, so its use can be problematic in PwPD. SSRIs (citalopram) and SNRIs (slow-release venlafaxine) have demonstrated antidepressant effects in clinical trials [157, 193] and may have less side effects compared with TCAs. The combination of an SSRI with an irreversible monoamine oxidase-B (MAO-B) inhibitor like selegiline is not recommended because of the risk of developing serotonergic syndrome [194]. The use of trazodone as a dual-acting serotonergic agent, with its presynaptic inhibition of serotonin reuptake and postsynaptic blockade of 5-HT2A receptors, strong affinity for central alpha-1 receptors, weak antagonistic affinity for H1 receptors, and lack of anticholinergic activity [195], also appears feasible in the treatment of depression in PwPD. Trazodone has a sedative effect that may be beneficial in PwPD with sleep problems. Given the limited clinical data, though, trazodone is a second-line recommendation [196, 197].

Unchanged from the previous German PD guidelines from 2016 [181], the authors recommend venlafaxine and desipramine for the treatment of major depression in PwPD, as these drugs have been clinically investigated and have acceptable safety profiles. The authors recommend treating moderate depressive disorders in PwPD according to the clinical phenotype, distinguishing between PwPD with psychomotor retardation, agitation, or the coexistence of other influencing factors like pain, salivation, cognitive impairment, and sleep disturbances.

There were no trials that investigated the effect of medication on anxiety in PwPD without underlying depression. Clinical trials in psychiatric populations have shown a beneficial effect of citalopram on anxiety disorders [198, 199]. Although the clinical symptoms of PwPD do not correspond to those of a psychiatric population, a trial of citalopram for the treatment of anxiety symptoms can be recommended for PwPD.

Mirtazepine is a noradrenergic and specific 5-HT1A-mediated serotonergic as well as 5-HT2 and 5-HT3 receptor blocking substance. Numerous in vitro and in vivo investigations have demonstrated not only the antioxidant and anti-inflammatory properties of Mirtazapine but also anti‑inflammatory and anti‑apoptotic effects. Due to the pathophysiology of PD, this might be of special interest and be advantageous in this target group. Because Mirtazapine can trigger REM sleep behavior disorder (RBD) likely due to its lacking anticholinergic activity it should not be applied in concomitant RBD [200], which is common in PD. The HT2a/c antagonistic effect is likely to be advantageous in PD patients with psychotic symptoms. Mirtazapine has antihistamine sedative properties due to its histamine H1 receptor blocking effect and shows a good safety and tolerability profile [201–203]. Therefore, it is broadly and preferentially used by Parkinson specialists in Germany in depressed PwPD especially with comorbid insomnia [204] and without RBD.

Overall, four reviews suggest Mirtazapine in PwPD and moderate depression with concomitant night-time agitation, anxiety, restlessness, sleep disturbances/insomnia and/or hallucinations/psychosis [197, 203, 205, 206]. It has to be considered, that these suggestions are based on the pharmacological profile of Mirtazapine and with respect to the recommendation for PwPD with depression and psychosis on four case reports [207–210].

The following recommendations not only involved an evaluation of the results of RCTs but also considered the efficacy profiles of antidepressants, which have been reported in clinical studies of psychiatric patient populations. The authors evaluated these findings within the clinical context of PD.

**Table:** 

*Recommendations for non-dopaminergic drug therapy to treat depressive disorders (updated)*
Severe depression might be treated with
- Venlafaxine 75–225 mg
- Desipramine 25–200 mg
Moderate depression might be treated according to its clinical phenotype
Dominance of psychomotor retardation
- Venlafaxine 75–150 mg
- Citalopram 20–40 mg
- Sertraline 50–100 mg
Dominance of agitation, anxiety, restlessness, or sleep disturbances
- Mirtazapine 15–45 mg (not for REM sleep behavior disorder)
- Trazodone 100–200 mg
Comorbidities of pain, salivation, cognitive impairment, or sleep disturbances
- Amitriptyline 10–75 mg retard
Level of consensus: 100%, strong consensus
*Recommendations for non-dopaminergic drug therapy to treat anxiety or panic disorders (new)*
Anxiety or panic disorder
- Non-dopaminergic drugs to treat anxiety and panic disorders have not been sufficiently tested
Citalopram 20–40 mg might be used
Level of consensus: 95%, strong consensus

#### Apathy

Safinamide works by inhibiting MAO-B and abnormal glutamate release. The efficacy of safinamide in non-motor PD symptoms, including affective disorders, was investigated in a post-hoc analysis of data from two RCTs. Safinamide showed a significant positive effect on quality of life (tested using the Parkinson’s Disease Questionnaire’s [PDQ-39] emotional well-being subscale) and depressive symptoms (tested using the GRID–Hamiliton Depression Rating Scale [GRID–HAM-D]) at 6 and 24 months compared to a placebo [211, 212]. A prospective, single-armed, open-label (uncontrolled) study of the efficacy of safinamide on non-motor symptoms showed a significant improvement in mood and apathy symptoms over 6 months among 44 PwPD, with good overall tolerability [212]. Whether this effect is driven by a non-dopaminergic modulation of glutamatergic hyperactivity or by primarily dopaminergic reversible MAO-B inhibition remains unanswered, though.

#### Anhedonia

There were no RCTs on the treatment of anhedonia.

#### Fatigue

Methylphenidate (30 mg per day) showed a significant reduction in the two primary outcomes (tested using the FSS and Multidimensional Fatigue Inventory) of an RCT with 36 PwPD [213]. This positive result was tempered by two factors. First, a group analysis (verum vs. placebo) was not performed, and second, no statistical correction for multiple outcomes was performed. The effect of modafinil on fatigue symptoms in PD was investigated in two RCTs [214, 215]. Given the inclusion of small sample sizes (*n* = 19 and *n* = 13) and undefined primary outcomes, modafinil could not be recommended [181, 211]. Safinamide was tested as an add-on therapy in 39 PwPD to treat fatigue over 24 weeks. The results showed an improvement in fatigue compared with the baseline using the FSS and Parkinson’s Fatigue Scale-16 (PFS-16). Specifically, 46% (FSS) and 41% (PFS-16) of PwPD were fatigue-free at the end of the study [216].

**Table:** 

*Recommendations for non-dopaminergic drug therapy to treat apathy, anhedonia, and fatigue (new)*
Apathy might be treated using
- Venlafaxine 75–225 mg retard
- Nortriptyline 25–150 mg
Level of consensus: 96.2%, strong consensus
Anhedonia
- There is no evidence for the use of non-dopaminergic medication for the treatment of anhedonia in PwPD
Level of consensus: 100%, strong consensus
Fatigue
- Non-dopaminergic drugs have not been sufficiently tested
- Modafinil 100–200 mg or safinamide 100 mg might be used
Level of consensus: 96.2%, strong consensus

An overview of the recommendations for the non-dopaminergic treatment of affective disorders in PwPD is given in Table 4.

**Table 4:** Overview of recommendations for non-dopaminergic treatment of affective disorders in PwPD

	Venlafaxine	Desipramine	Citaloprame	Sertraline	Mirtazapine	Trazodone	Amitriptyline	Nortriptyline	Modafinile	Safinamide
Major depression	+	+	–	–	–	–	–	–	–	–
Moderate depression with comorbidity of…	+ Psychomotor retardation	–	+ Psychomotor retardation	+ Psychomotor retardation	+ Agitation	+ Agitation	+ Sleep disturbance, hypersalivation or pain only in cognitively intact patients	–	–	–
Apathy	+	–	–	–	–	–	–	+	–	–
Anxiety	–	–	+	–	–	–	–	–	–	–
Fatigue	–	–	–	–	–	–	–	–	+	+

+ might be offered; – no recommendation

## Discussion

This guideline article summarized evidence for and expert opinions on diagnostic and therapeutic practices in the management of cognitive impairment and affective disorders in PwPD, and the derived recommendations for the German health care system were outlined.

Since the diagnosis of cognitive impairment in PwPD should be based on assessments with sufficient diagnostic properties [15], these guidelines included recommendations for suitable German global cognitive scales and tests to assess specific cognitive functions, as well as ADL assessment measures, to support the diagnosis of PD–MCI and PDD. However, more studies are needed to evaluate the discriminatory diagnostic power of (the German versions of) the instruments. To treat PD–MCI, cognitive training and physical interventions are recommended, whereas currently available evidence does not show that pharmacological treatment comprising AChEIs and memantine is sufficiently efficacious. In general, double-blinded RCTs with people with PD–MCI in multicenter studies should be performed to obtain more knowledge about the optimized intensity and duration of cognitive training. For PDD therapy, the AChEI rivastigmine and the off-label use of donepezil, as well as cognitive stimulation and reminiscence therapy as additional non-pharmacological treatment approaches, are recommended. Notably, there is currently a lack of therapists and prescriptions for the non-pharmacological treatment of cognitive symptoms in PwPD in Germany. The potential of food supplements and herbal therapeutics as a treatment option for cognitive impairment in PwPD needs further investigation in double-blinded, placebo-controlled RCTs. To date, there is a lack of knowledge about to what extent non-pharmacological and pharmacological interventions can prevent or delay a conversion to PD–MCI and PDD and, therefore, whether these interventions are effective to modulate cognitive deterioration in PwPD.

The guidelines provide a new outlook for obtaining medical histories and using scales to diagnose and track affective disorders in PwPD. Further, in addition to the update to dopaminergic and non-dopaminergic treatments, non-pharmacological treatment options like CBT and physical interventions could now be included and recommended. Despite their prevalence and impact on the quality of life of PwPD and that of their relatives, affective disorders are largely underdiagnosed [4] and, consequently, undertreated. Therefore, these recommendations may serve as the basis for implementing this largely neglected aspect into clinical diagnostic and treatment routines for PwPD. Here, the limited availability of psychotherapists and providers of physical interventions for PwPD in Germany is a challenge. Furthermore, for most non-pharmacological interventions, evidence is still too rare to derive more detailed recommendations regarding, e.g., duration and frequency of the treatment. Defining these treatment regimes with best effects for specific outcomes should be subject to further studies and could thus be integrated in future guidelines. This also holds true for further intervention types with potential benefits which could not be considered here.

No data are currently available on the implementation of the outlined recommendations in Germany. At best, guideline recommendations are based on the results of RCTs, but they cannot cover all the factors that influence the treatment of patients. Therefore, there are often medical conditions in the prevention, diagnosis, and treatment stages that cause the practitioner to deviate from the guidelines. The following sections outline some of the daily considerations for practice, in addition to guideline issues.

When diagnosing cognitive impairment in PwPD, competing causes must be considered on an individual basis. These include coexisting vascular cerebral damage, Alzheimer ‘s disease, cognitive disorders as a sign of depression, vitamin deficiency (B12, folic acid), substance abuse, metabolic disorders, and normal pressure hydrocephalus. In addition to a medical history, a physical examination, and neuropsychological testing, serological and biochemical tests of the blood are often required, in addition to the infrequent need for cerebrospinal fluid analysis. The anticholinergic activity of both PD and non-PD medications is of particular importance given its negative effect on cognitive functioning [217]. This is often the case with drugs used to treat non-motor symptoms (antidepressants, antipsychotics, bladder dysfunction therapy) and drugs not associated with PD therapy (antihistamines, anticonvulsants, opioids, glucocorticoids). The potency of drugs’ cognitive side effects is determined by the affinity of the muscarinic receptor type, pharmacokinetics, lipophilicity, and thus, potency of its crossover of the blood–brain barrier. A review summarizing drugs with an anticholinergic effect that also negatively affect cognition is available [218].

With neuropsychiatric symptoms, several differential diagnoses need to be considered, including anemia, hypothyroidism, metabolic disorders, or vitamin deficiencies. Sleep disorders should also be considered in differential diagnoses, especially with apathy and fatigue. The sometimes difficult clinical task is then to distinguish cognitive fatigue from physical fatigue. Sleep disturbances may lead to both cognitive and physical symptoms of fatigue. Since there is little correlation between subjective and objective measures of sleep in PwPD [219], the patient ‘s medical history is often not sufficiently informative.

Future research will have to investigate the utilization and feasibility of the suggested guidelines in clinical practice. Due to limited time and personnel resources and/or a lack of awareness of cognitive and affective disorders in PwPD, both the use of standardized diagnoses of these symptoms and prescriptions of (especially non-pharmacological) therapies constitute a major challenge.

## Supplementary Information

Below is the link to the electronic supplementary material.

Supplementary file1 (DOCX 57 KB)

## Data Availability

Systematic literature search data will be shared upon request by any qualified investigator. Data sharing requests should be made in writing to Professor Dr Inga Liepelt-Scarfone (chapter “Cognitive impairment,” inga.liepelt uni-tuebingen.de, Eberhard–Karls University Tübingen) and Professor Dr Elke Kalbe (chapter “Affective disorders,” elke.kalbe uk-koeln.de, University of Cologne) and require a formal data sharing agreement with approval from the respective university and the guideline office of the DGN. Data sharing agreements must include details on how the data will be stored, who will have access to the data and the intended use of the data, and agreements about the allocation of intellectual property.

## References

[1] Macías-García P, Rashid-López R, Cruz-Gómez ÁJ, Lozano-Soto E, Sanmartino F, Espinosa-Rosso R, González-Rosa JJ (2022) Neuropsychiatric symptoms in clinically defined Parkinson’s disease: an updated review of literature. Behav Neurol 2022:1213393. 10.1155/2022/1213393 35586201 10.1155/2022/1213393 PMC9110237

[2] Aarsland D, Batzu L, Halliday GM, Geurtsen GJ, Ballard C, Ray Chaudhuri K, Weintraub D (2021) Parkinson disease-associated cognitive impairment. Nat Rev Dis Primers 7:47. 10.1038/s41572-021-00280-3 34210995 10.1038/s41572-021-00280-3

[3] Duncan GW, Khoo TK, Yarnall AJ, O’Brien JT, Coleman SY, Brooks DJ, Barker RA, Burn DJ (2014) Health-related quality of life in early Parkinson’s disease: the impact of nonmotor symptoms. Mov Disord 29:195–202. 10.1002/mds.25664 24123307 10.1002/mds.25664

[4] Goodarzi Z, Mele B, Guo S, Hanson H, Jette N, Patten S, Pringsheim T, Holroyd-Leduc J (2016) Guidelines for dementia or Parkinson’s disease with depression or anxiety: a systematic review. BMC Neurol 16:244. 10.1186/s12883-016-0754-5 27887589 10.1186/s12883-016-0754-5 PMC5124305

[5] Deutsche Gesellschaft für Neurologie e.V. (DGN) (2023) S-2k-Leitlinie Parkinson-Krankheit. Version 8.1. https://register.awmf.org/de/leitlinien/detail/030-010. Accessed 9 Apr 2024

[6] Deutsche Gesellschaft für Psychiatrie und Psychotherapie, Psychosomatik und Nervenheilkunde e.V. (DGPPN) & Deutsche Gesellschaft für Neurologie e.V. (DGN) (2016) Leitlinie Demenzen Langversion—January 2016. https://register.awmf.org/assets/guidelines/038-013l_S3-Demenzen-2016-07.pdf. Accessed 9 April 2024

[7] BÄK, KBV, AWMF (2022) S3-Leiltinie Nationale VersorgungsLeiltinie Unipolare Depression. Version 3.2. https://register.awmf.org/de/leitlinien/detail/nvl-005. Accessed 9 Apr 2024

[8] Deutsche Gesellschaft für Psychosomatische Medizin und Ärztliche Psychotherapie e.V. (DGPM) (2021) S3-Leitlinie Behandlung von Angststörungen, Version 2.0. https://register.awmf.org/de/leitlinien/detail/051-028. Accessed 9 Apr 2024

[9] Aarsland D, Creese B, Politis M, Chaudhuri KR, Ffytche DH, Weintraub D, Ballard C (2017) Cognitive decline in Parkinson disease. Nat Rev Neurol 13:217–231. 10.1038/nrneurol.2017.27 28257128 10.1038/nrneurol.2017.27 PMC5643027

[10] Hely MA, Reid WG, Adena MA, Halliday GM, Morris JG (2008) The Sydney multicenter study of Parkinson’s disease: the inevitability of dementia at 20 years. Mov Disord 23:837–844. 10.1002/mds.21956 18307261 10.1002/mds.21956

[11] Bjornestad A, Pedersen KF, Tysnes OB, Alves G (2017) Clinical milestones in Parkinson’s disease: a 7-year population-based incident cohort study. Parkinsonism Relat Disord 42:28–33. 10.1016/j.parkreldis.2017.05.025 28578818 10.1016/j.parkreldis.2017.05.025

[12] Svenningsson P, Westman E, Ballard C, Aarsland D (2012) Cognitive impairment in patients with Parkinson’s disease: diagnosis, biomarkers, and treatment. Lancet Neurol 11:697–707. 10.1016/S1474-4422(12)70152-7 22814541 10.1016/S1474-4422(12)70152-7

[13] Saredakis D, Collins-Praino LE, Gutteridge DS, Stephan BCM, Keage HAD (2019) Conversion to MCI and dementia in Parkinson’s disease: a systematic review and meta-analysis. Parkinsonism Relat Disord 65:20–31. 10.1016/j.parkreldis.2019.04.020 31109727 10.1016/j.parkreldis.2019.04.020

[14] Lerche S, Wurster I, Roben B, Machetanz G, Zimmermann M, Bernhard F, Stransky E, Deuschle C, Schulte C, Hansson O, Zetterberg H, Gasser T, Berg D, Maetzler W, Brockmann K (2019) Parkinson’s disease: evolution of cognitive impairment and CSF Abeta1-42 profiles in a prospective longitudinal study. J Neurol Neurosurg Psychiatry 90(2):165–170. 10.1136/jnnp-2018-318956 30254084 10.1136/jnnp-2018-318956

[15] Litvan I, Goldman JG, Troster AI, Schmand BA, Weintraub D, Petersen RC, Mollenhauer B, Adler CH, Marder K, Williams-Gray CH, Aarsland D, Kulisevsky J, Rodriguez-Oroz MC, Burn DJ, Barker RA, Emre M (2012) Diagnostic criteria for mild cognitive impairment in Parkinson’s disease: Movement Disorder Society Task Force guidelines. Mov Disord 27:349–356. 10.1002/mds.24893 22275317 10.1002/mds.24893 PMC3641655

[16] Dubois B, Burn D, Goetz C, Aarsland D, Brown RG, Broe GA, Dickson D, Duyckaerts C, Cummings J, Gauthier S, Korczyn A, Lees A, Levy R, Litvan I, Mizuno Y, McKeith IG, Olanow CW, Poewe W, Sampaio C, Tolosa E, Emre M (2007) Diagnostic procedures for Parkinson’s disease dementia: recommendations from the movement disorder society task force. Mov Disord 22:2314–2324. 10.1002/mds.21844 18098298 10.1002/mds.21844

[17] Litvan I, Aarsland D, Adler H, Goldman JG, Kulisevsky J, Mollenhauer B, Rodriguez-Oroz MC, Tröster AI, Weintraub D (2011) MDS Task Force on mild cognitive impairment in Parkinson’s disease: critical review of PD-MCI. Mov Disord 26:1814–1824. 10.1002/mds.23823 21661055 10.1002/mds.23823 PMC3181006

[18] Emre M, Aarsland D, Brown R, Burn DJ, Duyckaerts C, Mizuno Y, Broe GA, Cummings J, Dickson DW, Gauthier S, Goldman J, Goetz C, Korczyn A, Lees A, Levy R, Litvan I, McKeith I, Olanow W, Poewe W, Quinn N, Sampaio C, Tolosa E, Dubois B (2007) Clinical diagnostic criteria for dementia associated with Parkinson’s disease. Mov Disord 22:689–1707. 10.1002/mds.21507 10.1002/mds.21507 17542011

[19] Gibb WR, Lees AJ (1988) The relevance of the Lewy body to the pathogenesis of idiopathic Parkinson’s disease. J Neurol Neurosurg Psychiatry 51:745–752. 10.1136/jnnp.51.6.745 2841426 10.1136/jnnp.51.6.745 PMC1033142

[20] Postuma RB, Berg D, Stern M, Poewe W, Olanow CW, Oertel W, Obeso J, Marek K, Litvan I, Lang AE, Halliday G, Goetz CG, Gasser T, Dubois B, Chan P, Bloem BR, Adler CH, Deuschl G (2015) MDS clinical diagnostic criteria for Parkinson’s disease. Mov Disord 30(12):1591–1601. 10.1002/mds.26424 26474316 10.1002/mds.26424

[21] Skorvanek M, Goldman JG, Jahanshahi M, Marras C, Rektorova I, Schmand B, van Duijn E, Goetz CG, Weintraub D, Stebbins GT, Martinez-Martin P, members of the MDS Rating Scales Review Committee (2018) Global scales for cognitive screening in Parkinson’s disease: critique and recommendations. Mov Disord 33:208–218. 10.1002/mds.27233 29168899 10.1002/mds.27233

[22] Sulzer P, Becker S, Maetzler W, Kalbe E, van Nueten L, Timmers M, Machetanz G, Streffer J, Salvadore G, Scholz E, Tkaczynska Z, Brockmann K, Berg D, Liepelt-Scarfone I (2018) Validation of a novel Montreal Cognitive Assessment scoring algorithm in non-demented Parkinson’s disease patients. J Neurol 265:1976–1984. 10.1007/s00415-018-8942-4 29936665 10.1007/s00415-018-8942-4

[23] Fengler S, Kessler J, Timmermann L, Zapf A, Elben S, Wojtecki L, Tucha O, Kalbe E (2016) Screening for cognitive impairment in Parkinson’s disease: improving the diagnostic utility of the MoCA through subtest weighting. PLoS ONE 11:e0159318. 10.1371/journal.pone.0159318 27437705 10.1371/journal.pone.0159318 PMC4954721

[24] Dalrymple-Alford JC, MacAskill MR, Nakas CT, Livingston L, Graham C, Crucian GP, Melzer TR, Kirwan J, Keenan R, Wells S, Porter RJ, Watts R, Anderson TJ (2010) The MoCA: well-suited screen for cognitive impairment in Parkinson disease. Neurology 75:1717–1725. 10.1212/WNL.0b013e3181fc29c9 21060094 10.1212/WNL.0b013e3181fc29c9

[25] Boel JA, de Bie RM, Schmand BA, Dalrymple-Alford JC, Marras C, Adler CH, Goldmann JG (2022) Level I PD-MCI using global cognitive tests and the risk for Parkinson’s disease dementia. Mov Disord Clin Pract 9:479–483. 10.1002/mdc3.13451 35582313 10.1002/mdc3.13451 PMC9092740

[26] Thomann AE, Goettel N, Monsch RJ, Berres M, Jahn T, Steiner LA, Monsch AU (2018) The Montreal Cognitive Assessment: normative data from a German-speaking cohort and comparison with international normative samples. J Alzheimers Dis 64:643–655. 10.3233/JAD-180080 29945351 10.3233/JAD-180080 PMC6027948

[27] Pirogovsky E, Schiehser DM, Litvan I, Obtera KM, Burke MM, Lessig SL, Song DD, Liu L, Filoteo JV (2014) The utility of the Mattis Dementia Rating Scale in Parkinson’s disease mild cognitive impairment. Parkinsonism Relat Disord 20:627–631. 10.1016/j.parkreldis.2014.03.010 24709086 10.1016/j.parkreldis.2014.03.010

[28] Liepelt-Scarfone I, Gräber S, Kalbe E, Riedel O, Ringendahl H, Schmidt N, Witt K, Roeske S (2021) Empfehlungen zur neuropsychologischen Diagnostik beim Morbus Parkinson. Fortschr Neurol Psychiatr 89:363–373. 10.1055/a-1099-9332 33561875 10.1055/a-1099-9332

[29] Kalbe E, Calabrese P, Kohn N, Hilker R, Riedel O, Wittchen HU, Dodel R, Otto J, Ebersbach G, Kessler J (2008) Screening for cognitive deficits in Parkinson’s disease with the Parkinson neuropsychometric dementia assessment (PANDA) instrument. Parkinsonism Relat Disord 14:93–101. 10.1016/j.parkreldis.2007.06.008 17707678 10.1016/j.parkreldis.2007.06.008

[30] Hoops S, Nazem S, Siderowf AD, Duda JE, Xie SX, Stern MB, Weintraub D (2009) Validity of the MoCA and MMSE in the detection of MCI and dementia in Parkinson disease. Neurology 73:1738–1745. 10.1212/WNL.0b013e3181c34b47 19933974 10.1212/WNL.0b013e3181c34b47 PMC2788810

[31] Memory Clinic, Universitaere Altersmedizin FELIX PLATTER, Basel (2024) Die Neuropsychologische Testbatterie CERAD-Plus. https://www.memoryclinic.ch/de/main-navigation/neuropsychologen/cerad-plus. Accessed 9 Apr 2024

[32] Barton BR, Bernard B, Czernecki V, Goldman JG, Stebbins G, Dubois B, Goetz CG (2014) Comparison of the Movement Disorder Society Parkinson’s disease dementia criteria with neuropsychological testing. Mov Disord 29:1252–1257. 10.1002/mds.25902 24821679 10.1002/mds.25902

[33] Goldman JG, Holden S, Ouyang B, Bernard B, Goetz CG, Stebbins GT (2015) Diagnosing PD-MCI by MDS task force criteria: How many and which neuropsychological tests? Mov Disord 30:402–406. 10.1002/mds.26084 25449653 10.1002/mds.26084 PMC4357536

[34] Hoogland J, van Wanrooij LL, Boel JA, Goldman JG, Stebbins GT, Dalrymple-Alford JC, Marras C, Adler CH, Junque C, Pedersen KF, Mollenhauer B, Zabetian CP, Eslinger PJ, Lewis SJG, Wu RM, Klein M, Rodriguez-Oroz MC, Cammisuli DM, Barone P, Biundo R, de Bie RMA, Schmand BA, Tröster AI, Burn DJ, Litvan I, Filoteo JV, Geurtsen GJ, Weintraub D, IPMDS Study Group “Validation of Mild Cognitive Impairment in Parkinson Disease” (2018) Detecting mild cognitive deficits in Parkinson’s disease: comparison of neuropsychological tests. Mov Disord 33:1750–1759. 10.1002/mds.110 30216541 10.1002/mds.110 PMC6261669

[35] Bezdicek O, Stepankova H, Martinec Novakova L, Kopecek M (2016) Toward the processing speed theory of activities of daily living in healthy aging: normative data of the Functional Activities Questionnaire. Aging Clin Exp Res 28:239–247. 10.1007/s40520-015-0413-5 26231091 10.1007/s40520-015-0413-5

[36] Sikkes SA, de Lange-de Klerk ES, Pijnenburg YA, Scheltens P, Uitdehaag BM (2009) A systematic review of Instrumental Activities of Daily Living scales in dementia: room for improvement. J Neurol Neurosurg Psychiatry 80:7–12. 10.1136/jnnp.2008.155838 19091706 10.1136/jnnp.2008.155838

[37] Dujardin K, Dubois B, Tison F, Durif F, Bourdeix I, Péré JJ, Duhamel A, EXECUTIVE study group (2010) Parkinson’s disease dementia can be easily detected in routine clinical practice. Mov Disord 25:2769–2776. 10.1002/mds.23391 20925065 10.1002/mds.23391

[38] Giovannetti T, Britnell P, Brennan L, Siderowf A, Grossman M, Libon DJ, Bettcher BM, Rouzard F, Eppig J, Seidel GA (2012) Everyday action impairment in Parkinson’s disease dementia. J Int Neuropsychol Soc 18:787–798. 10.1017/S135561771200046X 22621995 10.1017/S135561771200046X PMC3648638

[39] Leroi I, McDonald K, Pantula H, Harbishettar V (2012) Cognitive impairment in Parkinson disease: impact on quality of life, disability, and caregiver burden. J Geriatr Psychiatry Neurol 25:208–214. 10.1177/0891988712464823 23172765 10.1177/0891988712464823

[40] Stella F, Banzato CEM, Quagliato EMAB, Viana MA, Christofoletti G (2008) Dementia and functional decline in patients with Parkinson’s disease. Dement Neuropsychol 2:96–101. 10.1590/S1980-57642009DN20200004 29213550 10.1590/S1980-57642009DN20200004 PMC5619577

[41] Wahle M, Häller S, Spiegel R (1996) Validation of the NOSGER (Nurses’ Observation Scale for Geriatric Patients): reliability and validity of a caregiver rating instrument. Int Psychogeriatr 8:525–547. 10.1017/s1041610296002864 9147168 10.1017/s1041610296002864

[42] Christ JB, Berger MF, Riedl E, Prakash D, Csoti I, Molt W, Gräber S, Brockmann K, Berg D, Liepelt-Scarfone I (2013) How precise are activities of daily living scales for the diagnosis of Parkinson’s disease dementia? A pilot study. Parkinsonism Relat Disord 19:371–374. 10.1016/j.parkreldis.2012.11.004 23231974 10.1016/j.parkreldis.2012.11.004

[43] Kulisevsky J, Fernández de Bobadilla R, Pagonabarraga J, Martínez-Horta S, Campolongo A, García-Sánchez C, Pascual-Sedano B, Ribosa-Nogué R, Villa-Bonomo C (2013) Measuring functional impact of cognitive impairment: validation of the Parkinson’s disease cognitive functional rating scale. Parkinsonism Relat Disord 19:812–817. 10.1016/j.parkreldis.2013.05.007 23773412 10.1016/j.parkreldis.2013.05.007

[44] Martinez-Martin P (2013) Dementia in Parkinson’s disease: usefulness of the pill questionnaire. Mov Disord 28:1832–1837. 10.1002/mds.25649 24123241 10.1002/mds.25649

[45] Bahar-Fuchs A, Martyr A, Goh AM, Sabates J, Clare L (2019) Cognitive training for people with mild to moderate dementia. Cochrane Database Syst Rev 3:CD013069. 10.1002/14651858.CD013069.pub2 30909318 10.1002/14651858.CD013069.pub2 PMC6433473

[46] Alloni A, Quaglini S, Panzarasa S, Sinforiani E, Bernini S (2018) Evaluation of an ontology-based system for computerized cognitive rehabilitation. Int J Med Inform 115:64–72. 10.1016/j.ijmedinf.2018.04.005 29779721 10.1016/j.ijmedinf.2018.04.005

[47] Bernini S, Panzarasa S, Barbieri M, Sinforiani E, Quaglini S, Tassorelli C, Bottiroli S (2021) A double-blind randomized controlled trial of the efficacy of cognitive training delivered using two different methods in mild cognitive impairment in Parkinson’s disease: preliminary report of benefits associated with the use of a computerized tool. Aging Clin Exp Res 33:1567–1575. 10.1007/s40520-020-01665-2 32895890 10.1007/s40520-020-01665-2

[48] De Luca R, Latella D, Maggio MG, Di Lorenzo G, Maresca G, Sciarrone F, Militi D, Bramanti P, Calabrò RS (2019) Computer assisted cognitive rehabilitation improves visuospatial and executive functions in Parkinson’s disease: preliminary results. NeuroRehabilitation 45:285–290. 10.3233/NRE-192789 31498141 10.3233/NRE-192789

[49] París AP, Saleta HG, de la Cruz Crespo Maraver M, Silvestre E, Freixa MG, Torrellas CP, Pont SA, Nadal MF, Garcia SA, Bartolomé MV, Fernández VL, Bayés AR (2011) Blind randomized controlled study of the efficacy of cognitive training in Parkinson’s disease. Mov Disord 26:1251–1258. 10.1002/mds.23688 21442659 10.1002/mds.23688

[50] Lawrence BJ, Gasson N, Johnson AR, Booth L, Loftus AM (2018) Cognitive training and transcranial direct current stimulation for mild cognitive impairment in Parkinson’s disease: a randomized controlled trial. Parkinsons Dis 2018:4318475. 10.1155/2018/4318475 29780572 10.1155/2018/4318475 PMC5892209

[51] Vlagsma TT, Duits AA, Dijkstra HT, van Laar T, Spikman JM (2020) Effectiveness of ReSET; a strategic executive treatment for executive dysfunctioning in patients with Parkinson’s disease. Neuropsychol Rehabil 30:67–84. 10.1080/09602011.2018.1452761 29566588 10.1080/09602011.2018.1452761

[52] Maggio MG, De Cola MC, Latella D, Maresca G, Finocchiaro C, La Rosa G, Cimino V, Sorbera C, Bramanti P, De Luca R, Calabrò RS (2018) What about the role of virtual reality in Parkinson disease’s cognitive rehabilitation? Preliminary findings from a randomized clinical trial. J Geriatr Psychiatry Neurol 31:312–318. 10.1177/0891988718807973 30360679 10.1177/0891988718807973

[53] van de Weijer SCF, Duits AA, Bloem BR, de Vries NM, Kessels RPC, Köhler S, Tissingh G, Kuijf ML (2020) Feasibility of a cognitive training game in Parkinson’s disease: the randomized Parkin’Play study. Eur Neurol 83:426–432. 10.1159/000509685 32756067 10.1159/000509685 PMC7592931

[54] Sousa NMF, Neri A, Brandi IV, Brucki SMD (2021) Impact of cognitive intervention on cognitive symptoms and quality of life in idiopathic Parkinson’s disease: a randomized and controlled study. Dement Neuropsychol 15:51–59. 10.1590/1980-57642021dn15-010005 33907597 10.1590/1980-57642021dn15-010005 PMC8049575

[55] Angelucci F, Peppe A, Carlesimo GA, Serafini F, Zabberoni S, Barban F, Shofany J, Caltagirone C, Costa A (2015) A pilot study on the effect of cognitive training on BDNF serum levels in individuals with Parkinson’s disease. Front Hum Neurosci 9:130. 10.3389/fnhum.2015.00130 25852518 10.3389/fnhum.2015.00130 PMC4360779

[56] Costa A, Peppe A, Serafini F, Zabberoni S, Barban F, Caltagirone C, Carlesimo GA (2014) Prospective memory performance of patients with Parkinson’s disease depends on shifting aptitude: evidence from cognitive rehabilitation. J Int Neuropsychol Soc 20(7):717–726. 10.1017/S1355617714000563 24967725 10.1017/S1355617714000563

[57] Kalbe E, Folkerts AK, Ophey A, Eggers C, Elben S, Dimenshteyn K, Sulzer P, Schulte C, Schmidt N, Schlenstedt C, Berg D, Witt K, Wojtecki L, Liepelt-Scarfone I (2020) Enhancement of executive functions but not memory by multidomain group cognitive training in patients with Parkinson’s disease and mild cognitive impairment: a multicenter randomized controlled trial. Parkinsons Dis 2020:4068706. 10.1155/2020/4068706 33312495 10.1155/2020/4068706 PMC7721510

[58] Leung IH, Walton CC, Hallock H, Lewis SJ, Valenzuela M, Lampit A (2015) Cognitive training in Parkinson disease: a systematic review and meta-analysis. Neurology 85:1843–1851. 10.1212/WNL.0000000000002145 26519540 10.1212/WNL.0000000000002145 PMC4662707

[59] Gavelin HM, Domellöf ME, Leung I, Neely AS, Launder NH, Nategh L, Finke C, Lampit A (2022) Computerized cognitive training in Parkinson’s disease: a systematic review and meta-analysis. Ageing Res Rev 80:101671. 10.1016/j.arr.2022.101671 35714854 10.1016/j.arr.2022.101671

[60] Lawrence BJ, Gasson N, Bucks RS, Troeung L, Loftus AM (2017) Cognitive training and noninvasive brain stimulation for cognition in Parkinson’s disease: a meta-analysis. Neurorehabil Neural Repair 31:597–606. 10.1177/1545968317712468 28583011 10.1177/1545968317712468

[61] Woods B, Kaur Rai H, Elliott E, Aguirre E, Orrel M, Spector A (2023) Cognitive stimulation to improve cognitive functioning in people with dementia. Cochrane Database Syst Rev 1:CD005562. 10.1002/14651858.CD005562.pub3 10.1002/14651858.CD005562.pub2 22336813

[62] Folkerts AK, Dorn ME, Roheger M, Maassen M, Koerts J, Tucha O, Altgassen M, Sack AT, Smit D, Haarmann L, Kalbe E (2018) Cognitive stimulation for individuals with Parkinson’s disease dementia living in long-term care: preliminary data from a randomized crossover pilot study. Parkinsons Dis 2018:8104673. 10.1155/2018/8104673 30631420 10.1155/2018/8104673 PMC6304852

[63] Woods B, O’Philbin L, Farrell EM, Spector AE, Orrell M (2018) Reminiscence therapy for dementia. Cochrane Database Syst Rev 3:CD001120. 10.1002/14651858.CD001120.pub3 29493789 10.1002/14651858.CD001120.pub3 PMC6494367

[64] Sofi F, Valecchi D, Bacci D, Abbate R, Gensini GF, Casini A, Macchi C (2011) Physical activity and risk of cognitive decline: a meta-analysis of prospective studies. J Intern Med 269:107–117. 10.1111/j.1365-2796.2010.02281.x 20831630 10.1111/j.1365-2796.2010.02281.x

[65] Hamer M, Chida Y (2009) Physical activity and risk of neurodegenerative disease: a systematic review of prospective evidence. Psychol Med 39:3–11. 10.1017/S0033291708003681 18570697 10.1017/S0033291708003681

[66] Zeng Y, Wang J, Cai X, Zhang X, Zhang J, Peng M, Xiao D, Ouyang H, Yan F (2023) Effects of physical activity interventions on executive function in older adults with dementia: a meta-analysis of randomized controlled trials. Geriatr Nurs 51:369–377. 10.1016/j.gerinurse.2023.04.012 37127013 10.1016/j.gerinurse.2023.04.012

[67] Folkerts AK, Ernst M, Gollan R, Cryns N, Monsef I, Skoetz N, Kalbe E (2024) Can physical exercise be considered as a promising enhancer of global cognition in people with Parkinson’s disease? Results of a systematic review and meta-analysis. J Parkinsons Dis. 10.3233/JPD-230343 38457150 10.3233/JPD-230343 PMC11380223

[68] Paul KC, Chuang YH, Shih IF, Keener A, Bordelon Y, Bronstein JM, Ritz B (2019) The association between lifestyle factors and Parkinson’s disease progression and mortality. Mov Disord 34:58–66. 10.1002/mds.27577 30653734 10.1002/mds.27577 PMC6544143

[69] Avenali M, Picascia M, Tassorelli C, Sinforiani E, Bernini S (2021) Evaluation of the efficacy of physical therapy on cognitive decline at 6-month follow-up in Parkinson disease patients with mild cognitive impairment: a randomized controlled trial. Aging Clin Exp Res 33:3275–3284. 10.1007/s40520-021-01865-4 33978924 10.1007/s40520-021-01865-4

[70] Silveira CRA, Roy EA, Intzandt BN, Almeida QJ (2018) Aerobic exercise is more effective than goal-based exercise for the treatment of cognition in Parkinson’s disease. Brain Cogn 122:1–8. 10.1016/j.bandc.2018.01.002 29331916 10.1016/j.bandc.2018.01.002

[71] Picelli A, Varalta V, Melotti C, Zatezalo V, Fonte C, Amato S, Saltuari L, Santamato A, Fiore P, Smania N (2016) Effects of treadmill training on cognitive and motor features of patients with mild to moderate Parkinson’s disease: a pilot, single-blind, randomized controlled trial. Funct Neurol 31:25–31. 10.11138/fneur/2016.31.1.025 27027891 10.11138/FNeur/2016.31.1.025 PMC4819815

[72] Altmann LJ, Stegemöller E, Hazamy AA, Wilson JP, Bowers D, Okun MS, Hass CJ (2016) Aerobic exercise improves mood, cognition, and language function in Parkinson’s disease: results of a controlled study. J Int Neuropsychol Soc 22:878–889. 10.1017/S135561771600076X 27655232 10.1017/S135561771600076X

[73] da Silva FC, Iop RDR, de Oliveira LC, Boll AM, de Alvarenga JGS, Gutierres Filho PJB, Bezerra A, de Melo LM (2018) Effects of physical exercise programs on cognitive function in Parkinson’s disease patients: a systematic review of randomized controlled trials of the last 10 years. PLoS ONE 13:e01931. 10.1371/journal.pone.0193113 10.1371/journal.pone.0193113 PMC5828448 29486000

[74] Inskip MJ, Mavros Y, Sachdev PS, Hausdorff JM, Hillel I, Singh MAF (2022) Promoting independence in Lewy body dementia through exercise: the PRIDE study. BMC Geriatr 22(1):650. 10.1186/s12877-022-03347-2 35945508 10.1186/s12877-022-03347-2 PMC9361699

[75] Tabak R, Aquije G, Fisher BE (2013) Aerobic exercise to improve executive function in Parkinson disease: a case series. J Neurol Phys Ther 37(2):58–64. 10.1097/NPT.0b013e31829219bc 23632453 10.1097/NPT.0b013e31829219bc

[76] Telenius EW, Engedal K, Bergland A (2015) Effect of a high-intensity exercise program on physical function and mental health in nursing home residents with dementia: an assessor blinded randomized controlled trial. PLoS ONE 10:e01261. 10.1371/journal.pone.0126102 10.1371/journal.pone.0126102 PMC4431827 25974049

[77] Phillips MCL, Murtagh DKJ, Gilbertson LJ, Asztely FJS, Lynch CDP (2018) Low-fat versus ketogenic diet in Parkinson’s disease: a pilot randomized controlled trial. Mov Disord 33:1306–1314. 10.1002/mds.27390 30098269 10.1002/mds.27390 PMC6175383

[78] Paknahad Z, Sheklabadi E, Derakhshan Y, Bagherniya M, Chitsaz A (2020) The effect of the Mediterranean diet on cognitive function in patients with Parkinson’s disease: a randomized clinical controlled trial. Complement Ther Med 50:102366. 10.1016/j.ctim.2020.102366 32444045 10.1016/j.ctim.2020.102366

[79] Li Z, Wang P, Yu Z, Cong Y, Sun H, Zhang J, Zhang J, Sun C, Zhang Y, Ju X (2015) The effect of creatine and coenzyme q10 combination therapy on mild cognitive impairment in Parkinson’s disease. Eur Neurol 73:205–211. 10.1159/000377676 25792086 10.1159/000377676

[80] Cho BH, Choi SM, Kim JT, Kim BC (2018) Association of coffee consumption and non-motor symptoms in drug-naïve, early-stage Parkinson’s disease. Parkinsonism Relat Disord 50:2–47. 10.1016/j.parkreldis.2018.02.016 10.1016/j.parkreldis.2018.02.016 29449185

[81] Cho BH, Choi SM, Kim BC (2019) Gender-dependent effect of coffee consumption on tremor severity in de novo Parkinson’s disease. BMC Neurol 19:194. 10.1186/s12883-019-1427-y 31412802 10.1186/s12883-019-1427-y PMC6693140

[82] Zhang C, Zang Y, Song Q, Li H, Hu L, Zhao W, Feng S, Gu F, Zhao F, Zhang C (2019) The efficacy of a “cocktail therapy” on Parkinson’s disease with dementia. Neuropsychiatr Dis Treat 15:1639–1647. 10.2147/NDT.S179453 31296990 10.2147/NDT.S179453 PMC6598748

[83] Hilker R, Thomas AV, Klein JC, Weisenbach S, Kalbe E, Burghaus L, Jacobs AH, Herholz K, Heiss WD (2005) Dementia in Parkinson disease: functional imaging of cholinergic and dopaminergic pathways. Neurology 65:1716–1722. 10.1212/01.wnl.0000191154.78131.f6 16344512 10.1212/01.wnl.0000191154.78131.f6

[84] Tiraboschi P, Hansen LA, Alford M, Sabbagh MN, Schoos B, Masliah E, Thal LJ, Corey-Bloom J (2000) Cholinergic dysfunction in diseases with Lewy bodies. Neurology 54:407–411. 10.1212/wnl.54.2.407 10668703 10.1212/wnl.54.2.407

[85] van der Zee S, Müller ML, Kanel P, van Laar T, Bohnen NI (2021) Cholinergic denervation patterns across cognitive domains in Parkinson’s disease. Mov Disord 36:642–650. 10.1002/mds.28360 33137238 10.1002/mds.28360 PMC7988481

[86] Gargouri F, Gallea C, Mongin M, Pyatigorskaya N, Valabregue R, Ewenczyk C, Sarazin M, Yahia-Cherif L, Vidailhet M, Lehéricy S (2019) Multimodal magnetic resonance imaging investigation of basal forebrain damage and cognitive deficits in Parkinson’s disease. Mov Disord 34:516–525. 10.1002/mds.27561 30536444 10.1002/mds.27561 PMC6590238

[87] van der Zee S, Kanel P, Gerritsen MJJ, Boertien JM, Slomp AC, Müller MLTM, Bohnen NI, Spikman JM, van Laar T (2022) Altered cholinergic innervation in de novo Parkinson’s disease with and without cognitive impairment. Mov Disord 37:713–723. 10.1002/mds.28913 35037719 10.1002/mds.28913 PMC9306739

[88] McShane R, Westby MJ, Roberts E, Minakaran N, Schneider L, Farrimond LE, Maayan N, Ware J, Debarros J (2019) Memantine for dementia. Cochrane Database Syst Rev 3:CD003154. 10.1002/14651858.CD003154.pub 30891742 10.1002/14651858.CD003154.pub6 PMC6425228

[89] Tsujihashi H, Matsuda H, Uejima S, Akiyama T, Kurita T (1989) Role of natural killer cells in bladder tumor. Eur Urol 16:444–449. 10.1159/000471637 2591428 10.1159/000471637

[90] Baik K, Kim SM, Jung JH, Lee YH, Chung SJ, Yoo HS, Ye BS (2021) Donepezil for mild cognitive impairment in Parkinson’s disease. Sci Rep 11(1):4734. 10.1038/s41598-021-84243-4 33637811 10.1038/s41598-021-84243-4 PMC7910590

[91] Sawada H, Oeda T, Kohsaka M, Umemura A, Tomita S, Park K, Mizoguchi K, Matsuo H, Hasegawa K, Fujimura H, Sugiyama H, Nakamura M, Kikuchi S, Yamamoto K, Fukuda T, Ito S, Goto M, Kiyohara K, Kawamura T (2018) Early use of donepezil against psychosis and cognitive decline in Parkinson’s disease: a randomised controlled trial for 2 years. J Neurol Neurosurg Psychiatry 89:1332–1340. 10.1136/jnnp-2018-318107 30076270 10.1136/jnnp-2018-318107 PMC6288700

[92] Sawada H, Oeda T, Kohsaka M, Tomita S, Umemura A, Park K, Yamamoto K, Kiyohara K (2021) Early-start vs. delayed-start donepezil against cognitive decline in Parkinson disease: a randomized clinical trial. Expert Opin Pharmacother 22:363–371. 10.1080/14656566.2020.1814255 32867552 10.1080/14656566.2020.1814255

[93] Kawashima S, RCIP-Nagoya Study Group, Matsukawa N (2022) Memantine for the patients with mild cognitive impairment in Parkinson’s disease: a pharmacological fMRI study. BMC Neurol 22:175. 10.1186/s12883-022-02699-x 35562711 10.1186/s12883-022-02699-x PMC9103297

[94] Rolinski M, Fox C, Maidment I, McShane R (2012) Cholinesterase inhibitors for dementia with Lewy bodies, Parkinson’s disease dementia and cognitive impairment in Parkinson’s disease. Cochrane Database Syst Rev 2012:CD006504. 10.1002/14651858.CD006504.pub2 22419314 10.1002/14651858.CD006504.pub2 PMC8985413

[95] Pagano G, Rengo G, Pasqualetti G, Femminella GD, Monzani F, Ferrara N, Tagliati M (2015) Cholinesterase inhibitors for Parkinson’s disease: a systematic review and meta-analysis. J Neurol Neurosurg Psychiatry 86:767–773. 10.1136/jnnp-2014-308764 25224676 10.1136/jnnp-2014-308764

[96] Wang HF, Yu JT, Tang SW, Jiang T, Tan CC, Meng XF, Wang C, Tan MS, Tan L (2015) Efficacy and safety of cholinesterase inhibitors and memantine in cognitive impairment in Parkinson’s disease, Parkinson’s disease dementia, and dementia with Lewy bodies: systematic review with meta-analysis and trial sequential analysis. J Neurol Neurosurg Psychiatry 86:135–143. 10.1136/jnnp-2014-307659 24828899 10.1136/jnnp-2014-307659

[97] Stinton C, McKeith I, Taylor JP, Lafortune L, Mioshi E, Mak E, Cambridge V, Mason J, Thomas A, O’Brien JT (2015) Pharmacological management of Lewy body dementia: a systematic review and meta-analysis. Am J Psychiatry 172:731–742. 10.1176/appi.ajp.2015.14121582 26085043 10.1176/appi.ajp.2015.14121582

[98] Matsunaga S, Kishi T, Yasue I, Iwata N (2016) Cholinesterase inhibitors for Lewy body disorders: a meta-analysis. Int J Neuropsychopharmacol 19:pyv086. 10.1093/ijnp/pyv086 10.1093/ijnp/pyv086 PMC4772820 26221005

[99] Brennan L, Pantelyat A, Duda JE, Morley JF, Weintraub D, Wilkinson JR, Moberg PJ (2016) Memantine and cognition in Parkinson’s disease dementia/dementia with Lewy bodies: a meta-analysis. Mov Disord Clin Pract 3:161–167. 10.1002/mdc3.12264 30363483 10.1002/mdc3.12264 PMC6178606

[100] Broen MP, Narayen NE, Kuijf ML, Dissanayaka NN, Leentjens AF (2016) Prevalence of anxiety in Parkinson’s disease: a systematic review and meta-analysis. Mov Disord 31:1125–1133. 10.1002/mds.26643 27125963 10.1002/mds.26643

[101] den Brok MG, van Dalen JW, van Gool WA, Moll van Charante EP, de Bie RM, Richard E (2015) Apathy in Parkinson’s disease: a systematic review and meta-analysis. Mov Disord 30:759–769. 10.1002/mds.26208 25787145 10.1002/mds.26208

[102] Schrag A (2006) Quality of life and depression in Parkinson’s disease. J Neurol Sci 248:151–157. 10.1016/j.jns.2006.05.030 16797028 10.1016/j.jns.2006.05.030

[103] Burn DJ (2002) Beyond the iron mask: towards better recognition and treatment of depression associated with Parkinson’s disease. Mov Disord 17:445–454. 10.1002/mds.10114 12112190 10.1002/mds.10114

[104] Shulman LM, Taback RL, Rabinstein AA, Weiner WJ (2002) Non-recognition of depression and other non-motor symptoms in Parkinson’s disease. Parkinsonism Relat Disord 8:193–197. 10.1016/s1353-8020(01)00015-3 12039431 10.1016/s1353-8020(01)00015-3

[105] Richard IH, Kurlan R (1997) A survey of antidepressant drug use in Parkinson’s disease. Parkinson Study Group. Neurology 49:1168–1170. 10.1212/wnl.49.4.1168 9339713 10.1212/wnl.49.4.1168

[106] Weintraub D, Moberg PJ, Duda JE, Katz IR, Stern MB (2003) Recognition and treatment of depression in Parkinson’s disease. J Geriatr Psychiatry Neurol 16:178–183. 10.1177/0891988703256053 12967062 10.1177/0891988703256053

[107] Bundesinstitut für Arzneimittel und Medizinprodukte (2022) ICD-11 in Deutsch-Entwurfsfassung. https://www.bfarm.de/DE/Kodiersysteme/Klassifikationen/ICD/ICD-11/uebersetzung/_node.html;jsessionid=272C7E204316C0BDAB6ABCAC99C2E713.internet271. Accessed 13 June 2023

[108] Reiff J, Schmidt N, Riebe B, Breternitz R, Aldenhoff J, Deuschl G, Witt K (2011) Subthreshold depression in Parkinson’s disease. Mov Disord 26:1741–1744. 10.1002/mds.23699 21442660 10.1002/mds.23699

[109] Qin Z, Zhang L, Sun F, Liu H, Fang X, Chan P (2009) Depressive symptoms impacting on health-related quality of life in early Parkinson’s disease: results from Chinese l-dopa exposed cohort. Clin Neurol Neurosurg 111:733–737. 10.1016/j.clineuro.2009.07.001 19665835 10.1016/j.clineuro.2009.07.001

[110] Cong S, Xiang C, Zhang S, Zhang T, Wang H, Cong S (2022) Prevalence and clinical aspects of depression in Parkinson’s disease: a systematic review and meta-analysis of 129 studies. Neurosci Biobehav Rev 141:104749. 10.1016/j.neubiorev.2022.104749 35750224 10.1016/j.neubiorev.2022.104749

[111] Leentjens AF, Dujardin K, Marsh L, Martinez-Martin P, Richard IH, Starkstein SE, Weintraub D, Sampaio C, Poewe W, Rascol O, Stebbins GT, Goetz CG (2008) Apathy and anhedonia rating scales in Parkinson’s disease: critique and recommendations. Mov Disord 23:2004–2014. 10.1002/mds.22229 18709683 10.1002/mds.22229

[112] Nagayama H, Maeda T, Uchiyama T, Hashimoto M, Nomoto N, Kano O, Takahashi T, Terashi H, Hamada S, Hasegawa T, Hatano T, Takahashi T, Baba Y, Sengoku R, Watanabe H, Inoue M, Kadowaki T, Kaneko S, Shimura H, Kubo SI, Young Japanese Expert Group for Parkinson’s Disease and Movement Disorders: YJ-EXPANDS (2017) Anhedonia and its correlation with clinical aspects in Parkinson’s disease. J Neurol Sci 372:403–407. 10.1016/j.jns.2016.10.051 27839720 10.1016/j.jns.2016.10.051

[113] van der Velden RMJ, Broen MPG, Kuijf ML, Leentjens AFG (2018) Frequency of mood and anxiety fluctuations in Parkinson’s disease patients with motor fluctuations: a systematic review. Mov Disord 33:1521–1527. 10.1002/mds.27465 30225905 10.1002/mds.27465

[114] Berg P, Book K, Dinkel A, Henrich G, Marten-Mittag B, Mertens D, Ossner C, Volmer S, Herschbach P (2011) Progredienzangst bei chronischen Erkrankungen [Fear of progression in chronic diseases]. Psychother Psychosom Med Psychol 61:32–37. 10.1055/s-0030-1267927 21120791 10.1055/s-0030-1267927

[115] Folkerts AK, Haarmann L, Nielsen J, Saliger J, Eschweiler M, Karbe H, Allert N, Vida V, Trenkwalder C, Kruse A, Oelsner H, Ebersbach G, Kalbe E (2022) Fear of progression is determined by anxiety and self-efficacy but not disease-specific parameters in patients with Parkinson’s disease: preliminary data from a multicenter cross-sectional study. J Parkinsons Dis 12:2543–2553. 10.3233/JPD-223314 36189603 10.3233/JPD-223314

[116] Kluger BM, Herlofson K, Chou KL, Lou JS, Goetz CG, Lang AE, Weintraub D, Friedman J (2016) Parkinson’s disease-related fatigue: a case definition and recommendations for clinical research. Mov Disord 31:625–631. 10.1002/mds.26511 26879133 10.1002/mds.26511 PMC4863238

[117] Lou JS (2009) Physical and mental fatigue in Parkinson’s disease: epidemiology, pathophysiology and treatment. Drugs Aging 26:195–208. 10.2165/00002512-200926030-00002 19358616 10.2165/00002512-200926030-00002

[118] Metta V, Logishetty K, Martinez-Martin P, Gage HM, Schartau PE, Kaluarachchi TK, Martin A, Odin P, Barone P, Stocchi F, Antonini A, Chaudhuri KR (2011) The possible clinical predictors of fatigue in Parkinson’s disease: a study of 135 patients as part of international nonmotor scale validation project. Parkinsons Dis 2011:25271. 10.4061/2011/125271 10.4061/2011/125271 PMC3236421 22191065

[119] Friedman JH, Friedman H (2001) Fatigue in Parkinson’s disease: a 9-year follow-up. Mov Disord 16:1120–1122. 10.1002/mds.1201 11748745 10.1002/mds.1201

[120] Herlofson K, Kluger BM (2017) Fatigue in Parkinson’s disease. J Neurol Sci 374:38–41. 10.1016/j.jns.2016.12.061 28087059 10.1016/j.jns.2016.12.061

[121] Schwarz J, Odin P, Buhmann C, Csoti I, Jost W, Wüllner U, Storch A (2011) Depression in Parkinson’s disease. J Neurol 258:336–338. 10.1007/s00415-011-6048-3 10.1007/s00415-011-6048-3 21560065

[122] Brown RG, MacCarthy B, Gotham AM, Der GJ, Marsden CD (1988) Depression and disability in Parkinson’s disease: a follow-up of 132 cases. Psychol Med 18:49–55. 10.1017/s0033291700001872 3363044 10.1017/s0033291700001872

[123] Storch A, Ebersbach G, Fuchs G, Jost WH, Odin P, Reifschneider G, Bauer M (2008) Depression beim idiopathischen Parkinson-syndrom. Teil 1: epidemiologie, pathophysiologie, klinik und diagnostik [Depression in Parkinson’s disease. Part 1: epidemiology, signs and symptoms, pathophysiology and diagnosis]. Fortschr Neurol Psychiatr 76:715–724. 10.1055/s-2008-1038293 19012223 10.1055/s-2008-1038293

[124] Kummer A, Cardoso F, Teixeira AL (2009) Suicidal ideation in Parkinson’s disease. CNS Spectr 14:431–436. 10.1017/s109285290002040x 19890237 10.1017/s109285290002040x

[125] Lemke MR (2008) Depressive symptoms in Parkinson’s disease. Eur J Neurol 15:21–25. 10.1111/j.1468-1331.2008.02058.x 18353133 10.1111/j.1468-1331.2008.02058.x

[126] Pagonabarraga J, Kulisevsky J (2017) Apathy in Parkinson’s disease. Int Rev Neurobiol 133:657–678. 10.1016/bs.irn.2017.05.025 28802937 10.1016/bs.irn.2017.05.025

[127] Kluger BM (2017) Fatigue in Parkinson’s disease. Int Rev Neurobiol 133:743–768. 10.1016/bs.irn.2017.05.007 28802940 10.1016/bs.irn.2017.05.007

[128] Schrag A, Barone P, Brown RG, Leentjens AF, McDonald WM, Starkstein S, Weintraub D, Poewe W, Rascol O, Sampaio C, Stebbins GT, Goetz CG (2007) Depression rating scales in Parkinson’s disease: critique and recommendations. Mov Disord 22:1077–1092. 10.1002/mds.21333 17394234 10.1002/mds.21333 PMC2040268

[129] Nissenbaum H, Quinn NP, Brown RG, Toone B, Gotham AM, Marsden CD (1987) Mood swings associated with the ‘on-off’ phenomenon in Parkinson’s disease. Psychol Med 17:899–904. 10.1017/s0033291700000702 3432464 10.1017/s0033291700000702

[130] Raudino F (2001) Non motor off in Parkinson’s disease. Acta Neurol Scand 104:312–315. 10.1034/j.1600-0404.2001.00357.x 11696027 10.1034/j.1600-0404.2001.00357.x

[131] Zhang Q, Yang X, Song H, Jin Y (2020) Cognitive behavioral therapy for depression and anxiety of Parkinson’s disease: a systematic review and meta-analysis. Complement Ther Clin Pract 39:101111. 10.1016/j.ctcp.2020.101111 32379650 10.1016/j.ctcp.2020.101111

[132] Luo F, Ye M, Lv T, Hu B, Chen J, Yan J, Wang A, Chen F, He Z, Ding Z, Zhang J, Qian C, Liu Z (2021) Efficacy of cognitive behavioral therapy on mood disorders, sleep, fatigue, and quality of life in Parkinson’s disease: a systematic review and meta-analysis. Front Psychiatry 12:793804. 10.3389/fpsyt.2021.793804 34966313 10.3389/fpsyt.2021.793804 PMC8710613

[133] Xie CL, Wang XD, Chen J, Lin HZ, Chen YH, Pan JL, Wang WW (2015) A systematic review and meta-analysis of cognitive behavioral and psychodynamic therapy for depression in Parkinson’s disease patients. Neurol Sci 36:833–843. 10.1007/s10072-015-2118-0 25724804 10.1007/s10072-015-2118-0

[134] Bomasang-Layno E, Fadlon I, Murray AN, Himelhoch S (2015) Antidepressive treatments for Parkinson’s disease: a systematic review and meta-analysis. Parkinsonism Relat Disord 21:833–842. 10.1016/j.parkreldis.2015.04.018 26037457 10.1016/j.parkreldis.2015.04.018

[135] Tian J, Kang Y, Liu P, Yu H (2022) Effect of physical activity on depression in patients with Parkinson’s disease: a systematic review and meta-analysis. Int J Environ Res Public Health 19:6849. 10.3390/ijerph19116849 35682432 10.3390/ijerph19116849 PMC9180645

[136] Yang Y, Wang G, Zhang S, Wang H, Zhou W, Ren F, Liang H, Wu D, Ji X, Hashimoto M, Wei J (2022) Efficacy and evaluation of therapeutic exercises on adults with Parkinson’s disease: a systematic review and network meta-analysis. BMC Geriatr 22:813. 10.1186/s12877-022-03510-9 36271367 10.1186/s12877-022-03510-9 PMC9587576

[137] Gollan R, Ernst M, Lieker E, Caro-Valenzuela J, Monsef I, Dresen A, Roheger M, Skoetz N, Kalbe E, Folkerts AK (2022) Effects of resistance training on motor- and non-motor symptoms in patients with Parkinson’s disease: a systematic review and meta-analysis. J Parkinsons Dis 12:1783–1806. 10.3233/JPD-223252 35754291 10.3233/JPD-223252

[138] Wang LL, Sun CJ, Wang Y, Zhan TT, Yuan J, Niu CY, Yang J, Huang S, Cheng L (2022) Effects of dance therapy on non-motor symptoms in patients with Parkinson’s disease: a systematic review and meta-analysis. Aging Clin Exp Res 34:1201–1208. 10.1007/s40520-021-02030-7 35091970 10.1007/s40520-021-02030-7

[139] Zhang Q, Hu J, Wei L, Jia Y, Jin Y (2019) Effects of dance therapy on cognitive and mood symptoms in people with Parkinson’s disease: a systematic review and meta-analysis. Complement Ther Clin Pract 36:12–17. 10.1016/j.ctcp.2019.04.005 31383428 10.1016/j.ctcp.2019.04.005

[140] Wang K, Li K, Zhang P, Ge S, Wen X, Wu Z, Yao X, Jiao B, Sun P, Lv P, Lu L (2021) Mind-body exercises for non-motor symptoms of patients with Parkinson’s disease: a systematic review and meta-analysis. Front Aging Neurosci 13:770920. 10.3389/fnagi.2021.770920 36226304 10.3389/fnagi.2021.770920 PMC9549381

[141] Song R, Grabowska W, Park M, Osypiuk K, Vergara-Diaz GP, Bonato P, Hausdorff JM, Fox M, Sudarsky LR, Macklin E, Wayne PM (2017) The impact of Tai Chi and Qigong mind-body exercises on motor and non-motor function and quality of life in Parkinson’s disease: a systematic review and meta-analysis. Parkinsonism Relat Disord 41:3–13. 10.1016/j.parkreldis.2017.05.019 28602515 10.1016/j.parkreldis.2017.05.019 PMC5618798

[142] Jin X, Wang L, Liu S, Zhu L, Loprinzi PD, Fan X (2019) The impact of mind-body exercises on motor function, depressive symptoms, and quality of life in Parkinson’s disease: a systematic review and meta-analysis. Int J Environ Res Public Health 17:31. 10.3390/ijerph17010031 31861456 10.3390/ijerph17010031 PMC6981975

[143] Cugusi L, Solla P, Serpe R, Carzedda T, Piras L, Oggianu M, Gabba S, Di Blasio A, Bergamin M, Cannas A, Marrosu F, Mercuro G (2015) Effects of a Nordic Walking program on motor and non-motor symptoms, functional performance and body composition in patients with Parkinson’s disease. NeuroRehabilitation 37:245–254. 10.3233/NRE-151257 26484516 10.3233/NRE-151257

[144] Sacheli MA, Neva JL, Lakhani B, Murray DK, Vafai N, Shahinfard E, English C, McCormick S, Dinelle K, Neilson N, McKenzie J, Schulzer M, McKenzie DC, Appel-Cresswell S, McKeown MJ, Boyd LA, Sossi V, Stoessl AJ (2019) Exercise increases caudate dopamine release and ventral striatal activation in Parkinson’s disease. Mov Disord 34:1891–1900. 10.1002/mds.27865 31584222 10.1002/mds.27865

[145] Sajatovic M, Ridgel AL, Walter EM, Tatsuoka CM, Colón-Zimmermann K, Ramsey RK, Welter E, Gunzler SA, Whitney CM, Walter BL (2017) A randomized trial of individual versus group-format exercise and self-management in individuals with Parkinson’s disease and comorbid depression. Patient Prefer Adherence 11:965–973. 10.2147/PPA.S135551 28579759 10.2147/PPA.S135551 PMC5449131

[146] Abuoaf R, AlKaabi R, Mohamed Saleh A, Zerough U, Hartley T, van Niekerk SM, Khalil H, Morris LD (2023) The effect of physical exercise on anxiety in people with Parkinson’s disease: a systematic review of randomized control trials. NeuroRehabilitation 52:387–402. 10.3233/NRE-220264 37005897 10.3233/NRE-220264

[147] Herschbach P, Book K, Dinkel A, Berg P, Waadt S, Duran G, Engst-Hastreiter U, Henrich G (2010) Evaluation of two group therapies to reduce fear of progression in cancer patients. Support Care Cancer 18:471–479. 10.1007/s00520-009-0696-1 19865833 10.1007/s00520-009-0696-1

[148] Jiang C, Luo Y, Qu Y, Wang C, Li Z, Zhou J, Xu Z (2023) Pharmacological and behavioral interventions for fatigue in Parkinson’s disease: a meta-analysis of randomized controlled trials. J Geriatr Psychiatry Neurol 36:487–495. 10.1177/08919887231163291 36917786 10.1177/08919887231163291

[149] Bogosian A, Hurt CS, Hindle JV, McCracken LM, Vasconcelos e Sa DA, Axell S, Tapper K, Stevens J, Hirani PS, Salhab M, Ye W, Cubi-Molla P (2022) Acceptability and feasibility of a mindfulness intervention delivered via videoconferencing for people with Parkinson’s. J Geriatr Psychiatry Neurol 35:155–167. 10.1177/0891988720988901 33504245 10.1177/0891988720988901 PMC8678660

[150] Elbers RG, Verhoef J, van Wegen EE, Berendse HW, Kwakkel G (2015) Interventions for fatigue in Parkinson’s disease. Cochrane Database Syst Rev 2015:CD010925. 10.1002/14651858.CD010925.pub2 26447539 10.1002/14651858.CD010925.pub2 PMC9240814

[151] Folkerts AK, Nielsen J, Gollan R, Lansu A, Solfronk D, Monsef I, Ernst M, Nz S, Zeuner KE, Kalbe E (2023) Physical exercise as a potential treatment for fatigue in Parkinson’s disease? A systematic review and meta-analysis of pharmacological and non-pharmacological interventions. J Parkinsons Dis 13:659–679. 10.3233/JPD-225116 37334618 10.3233/JPD-225116 PMC10473113

[152] Witt K, Daniels C, Herzog J, Lorenz D, Volkmann J, Reiff J, Mehdorn M, Deuschl G, Krack P (2006) Differential effects of l-dopa and subthalamic stimulation on depressive symptoms and hedonic tone in Parkinson’s disease. J Neuropsychiatry Clin Neurosci 18:397–401. 10.1176/jnp.2006.18.3.397 16963590 10.1176/jnp.2006.18.3.397

[153] Barone P, Poewe W, Albrecht S, Debieuvre C, Massey D, Rascol O, Tolosa E, Weintraub D (2010) Pramipexole for the treatment of depressive symptoms in patients with Parkinson’s disease: a randomised, double-blind, placebo-controlled trial. Lancet Neurol 9:573–580. 10.1016/S1474-4422(10)70106-X 20452823 10.1016/S1474-4422(10)70106-X

[154] Ji N, Meng P, Xu B, Zhou X (2022) Efficacy and safety of pramipexole in Parkinson’s disease with anxiety or depression: a meta-analysis of randomised clinical trials. Am J Transl Res 14:1757–1764. 10.1111/ene.13303 35422951 PMC8991111

[155] Jiang DQ, Jiang LL, Wang Y, Li MX (2021) The role of pramipexole in the treatment of patients with depression and Parkinson’s disease: a meta-analysis of randomised controlled trials. Asian J Psychiatr 61:102691. 10.1016/j.ajp.2021.102691 33992852 10.1016/j.ajp.2021.102691

[156] Leentjens AF, Koester J, Fruh B, Shephard DT, Barone P, Houben JJ (2009) The effect of pramipexole on mood and motivational symptoms in Parkinson’s disease: a meta-analysis of placebo-controlled studies. Clin Ther 31:89–98. 10.1016/j.clinthera.2009.01.012 19243709 10.1016/j.clinthera.2009.01.012

[157] Ziaei E, Emami Ardestani P, Chitsaz A (2022) Comparison of pramipexole and citalopram in the treatment of depression in Parkinson’s disease: a randomised parallel-group trial. J Res Med Sci 27:55. 10.4103/jrms.jrms_790_21 36092482 10.4103/jrms.jrms_790_21 PMC9450243

[158] Chung SJ, Asgharnejad M, Bauer L, Ramirez F, Jeon B (2016) Evaluation of rotigotine transdermal patch for the treatment of depressive symptoms in patients with Parkinson’s disease. Expert Opin Pharmacother 17:1453–1461. 10.1080/14656566.2016.1202917 27322571 10.1080/14656566.2016.1202917

[159] Yan J, Ma H, Liu A, Huang J, Wu J, Yang J (2021) Efficacy and safety of rotigotine transdermal patch on neuropsychiatric symptoms of Parkinson’s disease: an updated meta-analysis and systematic review. Front Neurol 12:722892. 10.3389/fneur.2021.722892 34744967 10.3389/fneur.2021.722892 PMC8567083

[160] Wang HT, Wang L, He Y, Yu G (2018) Rotigotine transdermal patch for the treatment of neuropsychiatric symptoms in Parkinson’s disease: a meta-analysis of randomised placebo-controlled trials. J Neurol Sci 393:31–38. 10.1016/j.jns.2018.08.003 30099246 10.1016/j.jns.2018.08.003

[161] Ardouin C, Chéreau I, Llorca PM, Lhommée E, Durif F, Pollak P, Krack P (2009) Assessment of hyper- and hypodopaminergic behaviours in Parkinson’s disease. Rev Neurol 165:845–856. 10.1016/j.neurol.2009.06.003 19683776 10.1016/j.neurol.2009.06.003

[162] Ray Chaudhuri K, Martinez-Martin P, Antonini A, Brown RG, Friedman JH, Onofrj M, Surmann E, Ghys L, Trenkwalder C (2013) Rotigotine and specific non-motor symptoms of Parkinson’s disease: post hoc analysis of RECOVER. Parkinsonism Relat Disord 19:660–665. 10.1016/j.parkreldis.2013.02.018 23557594 10.1016/j.parkreldis.2013.02.018

[163] Lemke MR, Brecht HM, Koester J, Reichmann H (2006) Effects of the dopamine agonist pramipexole on depression, anhedonia and motor functioning in Parkinson’s disease. J Neurol Sci 248:266–270. 10.1016/j.jns.2006.05.024 16814808 10.1016/j.jns.2006.05.024

[164] Lemke MR, Brecht HM, Koester J, Kraus PH, Reichmann H (2005) Anhedonia, depression, and motor functioning in Parkinson’s disease during treatment with pramipexole. J Neuropsychiatry Clin Neurosci 17:214–220. 10.1176/jnp.17.2.214 15939976 10.1176/jnp.17.2.214

[165] Fabbri M, Barbosa R, Rascol O (2023) Off-time treatment options for Parkinson’s disease. Neurol Ther 12:391–424. 10.1007/s40120-022-00435-8 36633762 10.1007/s40120-022-00435-8 PMC10043092

[166] Czernecki V, Pillon B, Houeto JL, Pochon JB, Levy R, Dubois B (2002) Motivation, reward, and Parkinson’s disease: influence of dopatherapy. Neuropsychologia 40:2257–2267. 10.1016/s0028-3932(02)00108-2 12417456 10.1016/s0028-3932(02)00108-2

[167] Pérez-Pérez J, Pagonabarraga J, Martínez-Horta S, Fernández-Bobadilla R, Sierra S, Pascual-Sedano B, Gironell A, Kulisevsky J (2015) Head-to-head comparison of the neuropsychiatric effect of dopamine agonists in Parkinson’s disease: a prospective, cross-sectional study in non-demented patients. Drugs Aging 32:401–407. 10.1007/s40266-015-0264-y 25941103 10.1007/s40266-015-0264-y

[168] Thobois S, Lhommée E, Klinger H, Ardouin C, Schmitt E, Bichon A, Kistner A, Castrioto A, Xie J, Fraix V, Pelissier P, Chabardes S, Mertens P, Quesada JL, Bosson JL, Pollak P, Broussolle E, Krack P (2013) Parkinsonian apathy responds to dopaminergic stimulation of D2/D3 receptors with piribedil. Brain 136:1568–1577. 10.1093/brain/awt067 23543483 10.1093/brain/awt067

[169] Castrioto A, Thobois S, Anheim M, Quesada JL, Lhommée E, Klinger H, Bichon A, Schmitt E, Durif F, Azulay JP, Houeto JL, Longato N, Philipps C, Pelissier P, Broussolle E, Moro E, Tranchant C, Fraix V, Krack P (2020) A randomised controlled double-blind study of rotigotine on neuropsychiatric symptoms in de novo PD. NPJ Parkinsons Dis 6:41. 10.1038/s41531-020-00142-x 33319786 10.1038/s41531-020-00142-x PMC7738499

[170] Sawada H, Umemura A, Kohsaka M, Tomita S, Park K, Oeda T, Yamamoto K (2018) Pharmacological interventions for anxiety in Parkinson’s disease sufferers. Expert Opin Pharmacother 19:1071–1076. 10.1080/14656566.2018.1485650 29939821 10.1080/14656566.2018.1485650

[171] Prediger RD, Matheus FC, Schwarzbold ML, Lima MM, Vital MA (2012) Anxiety in Parkinson’s disease: a critical review of experimental and clinical studies. Neuropharmacology 62:115–124. 10.1016/j.neuropharm.2011.08.039 21903105 10.1016/j.neuropharm.2011.08.039

[172] Troeung L, Egan SJ, Gasson N (2013) A meta-analysis of randomised placebo-controlled treatment trials for depression and anxiety in Parkinson’s disease. PLoS ONE 8:e79510. 10.1371/journal.pone.0079510 24236141 10.1371/journal.pone.0079510 PMC3827386

[173] Schifitto G, Friedman JH, Oakes D, Shulman L, Comella CL, Marek K, Fahn S (2008) Fatigue in levodopa-naive subjects with Parkinson’s disease. Neurology 71:481–485. 10.1212/01.wnl.0000324862.29733.69 18695158 10.1212/01.wnl.0000324862.29733.69 PMC2937041

[174] Lou JS, Kearns G, Benice T, Oken B, Sexton G, Nutt J (2003) Levodopa improves physical fatigue in Parkinson’s disease: a double-blind, placebo-controlled, crossover study. Mov Disord 18:1108–1114. 10.1002/mds.10505 14534913 10.1002/mds.10505

[175] Hauser RA, Slawek J, Barone P, Dohin E, Surmann E, Asgharnejad M, Bauer L (2016) Evaluation of rotigotine transdermal patch for the treatment of apathy and motor symptoms in Parkinson’s disease. BMC Neurol 16:90. 10.1186/s12883-016-0610-7 27267880 10.1186/s12883-016-0610-7 PMC4895976

[176] Shannon KM, Bennett JP Jr, Friedman JH (1997) Efficacy of pramipexole, a novel dopamine agonist, as monotherapy in mild to moderate Parkinson’s disease. The Pramipexole Study Group. Neurology 49:724–728. 10.1212/wnl.49.3.724 9305331 10.1212/wnl.49.3.724

[177] Hauser RA, Schapira AH, Rascol O, Barone P, Mizuno Y, Salin L, Haaksma M, Juhel N, Poewe W (2010) Randomized, double-blind, multicenter evaluation of pramipexole extended release once daily in early Parkinson’s disease. Mov Disord 25:2542–2549. 10.1002/mds.23317 20669317 10.1002/mds.23317

[178] Maillet A, Météreau E, Tremblay L, Favre E, Klinger H, Lhommée E, Le Bars D, Castrioto A, Prange S, Sgambato V, Broussolle E, Krack P, Thobois S (2021) Serotonergic and dopaminergic lesions underlying parkinsonian neuropsychiatric signs. Mov Disord 36:2888–2900. 10.1002/mds.28722 34494685 10.1002/mds.28722

[179] Maillet A, Krack P, Lhommée E, Météreau E, Klinger H, Favre E, Le Bars D, Schmitt E, Bichon A, Pelissier P, Fraix V, Castrioto A, Sgambato-Faure V, Broussolle E, Tremblay L, Thobois S (2016) The prominent role of serotonergic degeneration in apathy, anxiety and depression in de novo Parkinson’s disease. Brain 139:2486–2502. 10.1093/brain/aww162 27538418 10.1093/brain/aww162

[180] Pagonabarraga J, Tinazzi M, Caccia C, Jost WH (2021) The role of glutamatergic neurotransmission in the motor and non-motor symptoms in Parkinson’s disease: clinical cases and a review of the literature. J Clin Neurosci 90:178–183. 10.1016/j.jocn.2021.05.056 34275546 10.1016/j.jocn.2021.05.056

[181] Deutsche Gesellschaft für Neurologie e.V. (DGN) (2016) S3-leitlinie idiopathisches Parkinson-syndrom. https://register.awmf.org/assets/guidelines/030-010k_S3_Parkinson_Syndrome_Idiopathisch_2016-06-abgelaufen.pdf. Accessed 9 Apr 2024

[182] Skapinakis P, Bakola E, Salanti G, Lewis G, Kyritsis AP, Mavreas V (2010) Efficacy and acceptability of selective serotonin reuptake inhibitors for the treatment of depression in Parkinson’s disease: a systematic review and meta-analysis of randomized controlled trials. BMC Neurol 10:49. 10.1186/1471-2377-10-49 20565960 10.1186/1471-2377-10-49 PMC2903535

[183] Fox SH, Katzenschlager R, Lim SY, Ravina B, Seppi K, Coelho M, Poewe W, Rascol O, Goetz CG, Sampaio C (2011) The movement disorder society evidence-based medicine review update: treatments for the motor symptoms of Parkinson’s disease. Mov Disord 26:2–41. 10.1002/mds.23829 22021173 10.1002/mds.23829

[184] Menza M, Dobkin RD, Marin H, Mark MH, Gara M, Buyske S, Bienfait K, Dicke A (2009) A controlled trial of antidepressants in patients with Parkinson disease and depression. Neurology 72:886–892. 10.1212/01.wnl.0000336340.89821.b3 19092112 10.1212/01.wnl.0000336340.89821.b3 PMC2677475

[185] Andersen J, Aabro E, Gulmann N, Hjelmsted A, Pedersen HE (1980) Anti-depressive treatment in Parkinson’s disease. A controlled trial of the effect of nortriptyline in patients with Parkinson’s disease treated with l-DOPA. Acta Neurol Scand 62:210–219. 10.1111/j.1600-0404.1980.tb03028.x 7010875 10.1111/j.1600-0404.1980.tb03028.x

[186] Devos D, Dujardin K, Poirot I, Moreau C, Cottencin O, Thomas P, Destée A, Bordet R, Defebvre L (2008) Comparison of desipramine and citalopram treatments for depression in Parkinson’s disease: a double-blind, randomized, placebo-controlled study. Mov Disord 23:850–857. 10.1002/mds.21966 18311826 10.1002/mds.21966

[187] Menza M, Dobkin RD, Marin H, Mark MH, Gara M, Buyske S, Bienfait K, Dicke A (2009) The impact of treatment of depression on quality of life, disability and relapse in patients with Parkinson’s disease. Mov Disord 24:1325–1332. 10.1002/mds.22586 19412944 10.1002/mds.22586 PMC2819360

[188] Antonini A, Tesei S, Zecchinelli A, Barone P, De Gaspari D, Canesi M, Sacilotto G, Meucci N, Mariani C, Pezzoli G (2006) Randomized study of sertraline and low-dose amitriptyline in patients with Parkinson’s disease and depression: effect on quality of life. Mov Disord 21:1119–1122. 10.1002/mds.20895 16637039 10.1002/mds.20895

[189] Meloni M, Puligheddu M, Carta M, Cannas A, Figorilli M, Defazio G (2020) Efficacy and safety of 5-hydroxytryptophan on depression and apathy in Parkinson’s disease: a preliminary finding. Eur J Neurol 27:779–786. 10.1111/ene.14179 32067288 10.1111/ene.14179

[190] da Silva TM, Munhoz RP, Alvarez C, Naliwaiko K, Kiss A, Andreatini R, Ferraz AC (2008) Depression in Parkinson’s disease: a double-blind, randomized, placebo-controlled pilot study of omega-3 fatty-acid supplementation. J Affect Disord 111:351–359. 10.1016/j.jad.2008.03.008 18485485 10.1016/j.jad.2008.03.008

[191] Gillman PK (2007) Tricyclic antidepressant pharmacology and therapeutic drug interactions updated. Br J Pharmacol 151:737–748. 10.1038/sj.bjp.0707253 17471183 10.1038/sj.bjp.0707253 PMC2014120

[192] McClure EW, Daniels RN (2021) Classics in chemical neuroscience: amitriptyline. ACS Chem Neurosci 12:354–362. 10.1021/acschemneuro.0c00467 33438398 10.1021/acschemneuro.0c00467

[193] Richard IH, McDermott MP, Kurlan R, Lyness JM, Como PG, Pearson N, Factor SA, Juncos J, Serrano Ramos C, Brodsky M, Manning C, Marsh L, Shulman L, Fernandez HH, Black KJ, Panisset M, Christine CW, Jiang W, Singer C, Horn S, Pfeiffer R, Rottenberg D, Slevin J, Elmer L, Press D, Hyson HC, McDonald W; SAD-PD Study Group (2012) A randomized, double-blind, placebo-controlled trial of antidepressants in Parkinson disease. Neurology 78:1229–1236. 10.1212/WNL.0b013e3182516244 22496199 10.1212/WNL.0b013e3182516244 PMC3324323

[194] Richard IH, Kurlan R, Tanner C, Factor S, Hubble J, Suchowersky O, Waters C (1997) Serotonin syndrome and the combined use of deprenyl and an antidepressant in Parkinson’s disease. Parkinson Study Group. Neurology 10748:1070–1077. 10.1212/wnl.48.4.1070 10.1212/wnl.48.4.1070 9109902

[195] Brogden RN, Heel RC, Speight TM, Avery GS (1981) Trazodone: a review of its pharmacological properties and therapeutic use in depression and anxiety. Drugs 21:401–429. 10.2165/00003495-198121060-00001 7018873 10.2165/00003495-198121060-00001

[196] Werneck AL, Rosso AL, Vincent MB (2009) The use of an antagonist 5-HT2a/c for depression and motor function in Parkinson’ disease. Arq Neuropsiquiatr 67:407–412. 10.1590/s0004-282x2009000300007 19623435 10.1590/s0004-282x2009000300007

[197] Peña E, Mata M, López-Manzanares L, Kurtis M, Eimil M, Martínez-Castrillo JC, Navas I, Posada IJ, Prieto C, Ruíz-Huete C, Vela L, Venegas B, on behalf of the movement disorder study group of the Neurological Association of Madrid (2018) Antidepressants in Parkinson’s disease. Recommendations by the movement disorder study group of the Neurological Association of Madrid. Neurologia 33:395–402. 10.1016/j.nrleng.2016.02.017 10.1016/j.nrl.2016.02.002 27004670

[198] Lenze EJ, Mulsant BH, Shear MK, Dew MA, Miller MD, Pollock BG, Houck P, Tracey B, Reynolds CF III (2005) Efficacy and tolerability of citalopram in the treatment of late-life anxiety disorders: results from an 8-week randomized, placebo-controlled trial. Am J Psychiatry 162:146–150. 10.1176/appi.ajp.162.1.146 15625213 10.1176/appi.ajp.162.1.146

[199] Varia I, Rauscher F (2002) Treatment of generalized anxiety disorder with citalopram. Int Clin Psychopharmacol 17:103–107. 10.1097/00004850-200205000-00002 11981350 10.1097/00004850-200205000-00002

[200] Onofrj M, Luciano AL, Thomas A, Iacono D, D’Andreamatteo G (2003) Mirtazapine induces REM sleep behavior disorder (RBD) in parkinsonism. Neurology 60:113–115. 10.1212/01.wnl.0000042084.03066.c0 12525729 10.1212/01.wnl.0000042084.03066.c0

[201] Anttila SA, Leinonen EV (2001) A review of the pharmacological and clinical profile of mirtazapine. CNS Drug Rev 7:249–264. 10.1111/j.1527-3458.2001.tb00198.x 11607047 10.1111/j.1527-3458.2001.tb00198.x PMC6494141

[202] Fawcett J, Barkin RL (1998) Review of the results from clinical studies on the efficacy, safety and tolerability of mirtazapine for the treatment of patients with major depression. J Affect Disord 51:267–285. 10.1016/s0165-0327(98)00224-9 10333982 10.1016/s0165-0327(98)00224-9

[203] Hassanein EHM, Althagafy HS, Baraka MA, Abd-Alhameed EK, Ibrahim IM (2024) Pharmacological update of mirtazapine: a narrative literature review. Naunyn Schmiedebergs Arch Pharmacol 397:2603–2619. 10.1007/s00210-023-02818-6 37943296 10.1007/s00210-023-02818-6 PMC11074035

[204] Kasten M, Hagenah J, Graf J, Lorwin A, Vollstedt EJ, Peters E, Katalinic A, Raspe H, Klein C (2013) Cohort profile: a population-based cohort to study non-motor symptoms in Parkinsonism (EPIPARK). Int J Epidemiol 42:128–128. 10.1093/ije/dys202 23257687 10.1093/ije/dys202

[205] De Rezende Costa FJ, Rosso ALZ, Maultasch H, Nicaretta DH, Vincent MB (2012) Depression in Parkinson’s disease: diagnosis and treatment. Arq Neuropsiquiatr 70:617–620. 10.1590/s0004-282x2012000800011 22899034 10.1590/s0004-282x2012000800011

[206] Sid-Otmane L, Huot P, Panisset M (2020) Effect of antidepressants on psychotic symptoms in Parkinson disease: a review of case reports and case series. Clin Neuropharm 43:61–65. 10.1097/WNF.0000000000000384 10.1097/WNF.0000000000000384 32217864

[207] Godschalx-Dekker JA, Siegers HP (2014) Reduction of parkinsonism and psychosis with mirtazapine: a case report. Pharmacopsychiatry 47:81–83. 10.1055/s-0034-1367014 24504487 10.1055/s-0034-1367014

[208] Tagai K, Nagata T, Shinagawa S, Tsuno N, Ozone M, Nakayama K (2013) Mirtazapine improves visual hallucinations in Parkinson’s disease: a case report. Psychogeriatrics 13:103–107. 10.1111/j.1479-8301.2012.00432.x 23909968 10.1111/j.1479-8301.2012.00432.x

[209] Nagata T, Shinagawa S, Tagai K, Nakayama K (2013) A case in which mirtazapine reduced auditory hallucinations in a patient with Parkinson disease. Psychogeriatrics 25:1199–1201. 10.1017/S1041610212002037 10.1017/S1041610212002037 23195073

[210] Normann C, Hesslinger B, Frauenknecht S, Berger M, Walden J (1997) Psychosis during chronic levodopa therapy triggered by the new antidepressive drug mirtazapine. Pharmacopsychiatry 30:263–265. 10.1055/s-2007-979504 9442549 10.1055/s-2007-979504

[211] Cattaneo C, Müller T, Bonizzoni E, Lazzeri G, Kottakis I, Keywood C (2017) Long-term effects of safinamide on mood fluctuations in Parkinson’s disease. J Parkinsons Dis 7:629–634. 10.3233/JPD-171143 28777756 10.3233/JPD-171143 PMC5676861

[212] Santos García D, Labandeira Guerra C, Yáñez Baña R, Cimas Hernando MI, Cabo López I, Paz Gonález JM, Alonso Losada MG, González Palmás MJ, Martínez Miró C (2021) Safinamide improves non-motor symptoms burden in Parkinson’s disease: an open-label prospective study. Brain Sci 11:316. 10.3390/brainsci11030316 33801565 10.3390/brainsci11030316 PMC7999475

[213] Mendonça DA, Menezes K, Jog MS (2007) Methylphenidate improves fatigue scores in Parkinson disease: a randomized controlled trial. Mov Disord 22:2070–2076. 10.1002/mds.21656 17674415 10.1002/mds.21656

[214] Lou JS, Dimitrova DM, Park BS, Johnson SC, Eaton R, Arnold G, Nutt JG (2009) Using modafinil to treat fatigue in Parkinson disease: a double-blind, placebo-controlled pilot study. Clin Neuropharmacol 32:305–310. 10.1097/WNF.0b013e3181aa916a 19620846 10.1097/WNF.0b013e3181aa916a

[215] Tyne HL, Taylor J, Baker GA, Steiger MJ (2010) Modafinil for Parkinson’s disease fatigue. J Neurol 257:452–456. 10.1007/s00415-009-5351-8 19842011 10.1007/s00415-009-5351-8

[216] Pauletti C, Locuratolo N, Mannarelli D, Maffucci A, Petritis A, Menini E, Fattapposta F (2023) Fatigue in fluctuating Parkinson’s disease patients: possible impact of safinamide. J Neural Transm (Vienna) 130:915–923. 10.1007/s00702-023-02654-1 37210459 10.1007/s00702-023-02654-1 PMC10293124

[217] Perez-Lloret S, Peralta MC, Barrantes FJ (2016) Pharmacotherapies for Parkinson’s disease symptoms related to cholinergic degeneration. Expert Opin Pharmacother 17:2405–2415. 10.1080/14656566.2016.1254189 27785919 10.1080/14656566.2016.1254189

[218] López-Álvarez J, Sevilla-Llewellyn-Jones J, Agüera-Ortiz L (2019) Anticholinergic drugs in geriatric psychopharmacology. Front Neurosci 13:1309. 10.3389/fnins.2019.01309 31866817 10.3389/fnins.2019.01309 PMC6908498

[219] Aitken D, Naismith SL, Terpening Z, Lewis SJ (2014) Dysfunctional sleep beliefs in Parkinson’s disease: relationships with subjective and objective sleep. J Clin Neurosci 21:1359–1363. 10.1016/j.jocn.2013.11.031 24661963 10.1016/j.jocn.2013.11.031

