# Supplementary material for: German Society of Neurology guidelines for the diagnosis and treatment of cognitive impairment and affective disorders in people with Parkinson’s disease: new spotlights on diagnostic procedures and non-pharmacological interventions
Source: J Neurol. 2024 Aug 9;271(11):7330–57. doi: 10.1007/s00415-024-12503-0 (PMC11561078; doi:10.1007/s00415-024-12503-0)
Supplement: Supplementary file 1 — Supplementary file1 (DOCX 57 KB) [file 415_2024_12503_MOESM1_ESM.docx]

**Supplementary Table 1.**

Overview of recommended German global cognitive screening scales with sufficient diagnostic accuracy to support DSM-V, ICD-10 or Level II cognitive diagnosis of Parkinson’s disease with mild cognitive impairment (PD-MCI) and Parkinson’s disease dementia (PDD).

| **Global cognitive scales** | **Reliability, validity & sensitivity of change** | | **Diagnostic accuracy for PD-MCI** | | | **Diagnostic accuracy for PDD** | | |
| --- | --- | --- | --- | --- | --- | --- | --- | --- |
|  | **Non-German version** | **German version** | **Cut-off** | **Sensitivity** | **Specificity** | **Cut-off** | **Sensitivity** | **Specificity** |
| Montreal Cognitive Assessment (MoCA) | Acceptable | Reliability & validity acceptable | <26 | 56-90% | 65-70-75% [1-3] | <21 | 81% | 95% [4] |
| Mattis-Dementia Rating Scale (MDRS) | Acceptable | Not defined | <140 | 77% | 65% [5] | <123 | 93% | 91% [6] |
| Scales for Outcomes in Parkinson’s disease-cognition (SCOPA-COG) | Validity acceptable | Not defined | <30 | 76% | 75% [1] | <23 | 80-85% | 87-96% [1, 7] |
| Addenbrooke’s Cognitive Examination I (ACE-I) | Validity acceptable | Validation in non-PD cohorts | 0-8 EY: <84  9-12 EY: <87 >12 EY: <91 | 85-86% | 57-63% [8] | 0-8 EY: <75  9-12 EY: <77 >12 EY: <81 | 82-90% | 74-93% [8] |
| Revised Addenbrooke’s Cognitive Examination (ACE-R) | Validity acceptable | Validation in non-PD cohorts | <78 | 59% | 81% [9] | - | - | - |
| Addenbrooke’s Cognitive Examination III (ACE-III) | Validity acceptable | Not defined | 0-8 EY: <84  9-12 EY: <86  >12 EY: <89 | 77-93% | 64-79% [8] | 0-8 EY: <71  9-12 EY: <78  >12 EY: <79 | 81-89% | 74-93% [8] |
| Mini Addenbrooke’s Cognitive Examination (Mini-ACE) | Validity acceptable | Not defined | 0-8 EY: <23  9-12 EY: <25  >12 EY: <26 | 72-85% | 51-80% [8] | 0-8 EY: <18  9-12 EY: <21  >12 EY: <22 | 72-91% | 75-92% [8] |
| Parkinson Neuropsychometric Dementia Assessment (PANDA) | Acceptable | Reliability & validity acceptable | <17* | 77% | 91% [10] | <15 | 72-97% | 71-100% [11-13] |
| Mini-Mental State Examination (MMSE) | Acceptable | Acceptable | - | - | - | <26 | 47% | 100% [14] |

* refers to a combined group of patients with cognitive impairment (PD-MCI and PDD); *Abbreviations:* EY: Education years.

**Supplementary Table 2.**

Individual studies on the treatment of depression in PwPD.

| **Study** | **Evidence level** | **Intervention** | **Daily dosage** | **Study design** | **Duration** | **N** | **Results** |
| --- | --- | --- | --- | --- | --- | --- | --- |
| Antonini 2006 [15]^a^ | 1- | Sertraline vs. Amitriptyline | Sertraline:  50 mg  Amitriptyline:  25 mg | Randomised, single-blinded (rater blinded) | 12 weeks | 31 | Significant decrease in HAMD-17 values in both groups. Significant improvement in quality of life (PDQ-39) with sertraline, but not with amitriptyline. No change in UPDRS III and MMSE in both groups.  CAVEAT: no true placebo arm |
| Barone 2006 [16]^a^ | 1- | Pramipexole vs. Sertraline | Pramipexole:  3 mg  Sertraline:  48 mg | Randomised, parallel group, open-label, single-blinded  (rater blinded) | 12 weeks | 67 | Significant decrease in HAM-D scores within both treatment groups after 12 weeks, no difference between the groups. 61% of patients on pramipexole and 27% of patients on sertraline showed HAM-D scores ≤ 7 after 12 weeks.  CAVEAT: no true placebo arm |
| da Silva 2008 [17]^a^ | 1+ | Omega 3 fatty acids vs. placebo | Eicosapentaenoic acid: 180 mg Docosahexaenoic acid: 120mg | Randomised, double-blind, placebo-controlled | 12 weeks | 31 | Significant reduction in depression scores in the Montgomery-Asberg Depression Rating Scale (MADRS) and CGI with omega-3 fatty acids. No significant difference in the BDI score. Response rate of 42% with omega 3 fatty acids compared to 6% with placebo |
| Devos 2008 [18] | 1++ | Despiramine vs.  Citaloprame vs. Placebo | Despiramine: 75 mg Citaloprame:  20 mg | Randomised, double-blind, placebo-controlled | 30 days | 48 | Significant improvement in MADRS score with despiramine and citalopram compared to placebo (primary endpoint). No significant worsening of cognitive and motor symptoms, but twice the rate of side effects with despiramine compared to the other two groups.  despiramine compared to the other two groups. No differences in the mean Hamilton Anxiety Rating Scale (HARS) scores. |
| Werneck 2009 [19] | 1- | Trazodone vs. no therapy | Trazodone:  50 mg 2 x/day. | Randomised, open-label, single-blinded (rater blinded) | 5 months | 22 | Significant improvement in depression (HAM-D) in the trazodone arm compared to the arm without therapy. Significant improvement in motor function (UPDRS) in the trazodone arm compared to the no treatment arm in depressed patients only.  CAVEAT: Depression was not an inclusion criterion, no true placebo arm. |
| Menza 2009 [20]^a^  Menza 2009 [21] | 1+/ 1- | Nortriptyline vs. Paroxe- tine CR vs. Placebo | Nortriptyline: 48,5 mg  Paroxetine controlled release (CR):  28,4 mg | Randomised, double-blind, placebo-controlled | 8 weeks +  16 weeks for blinded extension (to detect recurrence of depression)  recurrence of depression) | 52/ 20 | Superior effect of nortryptiline on HAMD-17 values compared to placebo. No significant difference between paroxetine and placebo. Higher response rates with nortriptyline compared to paroxetine and placebo. Advantage of nortriptyline over paroxetine and placebo in influencing sleep, anxiety and social functions.  In the observation phase of the blinded extension of the study, relapse was significantly more frequent with placebo (3 out of 5) than with paroxetine (0 out of 8) and nortriptyline (1 out of 7) |
| Barone 2010 [22]^a^ | 1++ | Pramipexoel vs. Placebo | 0,357–3 mg  (2,18 mg) | Randomised, double-blind, placebo-controlled | 12 weeks | 287 | Significant decrease in BDI score with pramipexole compared to placebo (primary endpoint). Path analysis with various regression models shows that a direct anti-depressive effect of pramipexole is responsible for 80% and an improvement in motor symptoms by pramipexole for 20% of the effect of pramipexole on the main target parameter (change in BDI) in the study. Significant advantage of pramipexole over placebo in the following secondary endpoints: CGI-I; GDS-15; EuroQuol and UPDRS II and III. |
| Richard 2012 [23] | 1++ | Paroxetine vs. Ven- lafaxine XR vs. Placebo | Paroxetine:  10-40 mg;  Venlafaxine XR:  37,5-225 mg | Randomised, double-blind, placebo-controlled | 12 weeks | 115 | Significant decrease in HAM-D score with paroxetine and venlafaxine compared to placebo. No significant difference between the two active treatment groups. |
| Meloni 2020 [24] | 1+ | 5-Hydroxytryptophane (5-HTP) vs. Placebo | 5-HTP 50 mg | Randomised, double-blind, placebo-controlled crossover | 4 weeks | 23 | Significant improvement in Hamilton Depression Rating Scale (HDRS) with 5-HTP compared to placebo. No significant difference between active treatment group and placebo with regard to the Beck Depression Inventory-II (BDI-II) and the Apathy Scale (AS). |

Adapted from 2016 S3 guidelines Parkinson’s disease [25]; ^a^ Studies that were included in the systematic review of Seppi et al. [26]; *Abbreviations:* BDI: Beck Depression Inventory; HAM-D: Hamilton Rating Scale for Depression; MADRS: Montgomery-Asberg Depression Rating Scale; GDS- 15: geriatric depression scale, MMSE: Mini Mental State Examination, CGI: Clinical Global Impression, UPDRS Unified Parkinson’s Disease Rating Scale, IDS- C: Inventory of Depressive Symptomatology-Clinician, PDQL: Parkinson’s disease and Quality of Life; CBT: Cognitive Behavioral Therapy.

**References of the Supplementary Material**

1. Dalrymple-Alford JC, MacAskill MR, Nakas CT, Livingston L, Graham C, Crucian GP, Melzer TR, Kirwan J, Keenan R, Wells S, Porter RJ, Watts R, Anderson TJ (2010) The MoCA: well-suited screen for cognitive impairment in Parkinson disease. Neurology 75(19):1717-1725. https://doi.org/10.1212/WNL.0b013e3181fc29c9
2. Fengler S, Kessler J, Timmermann L, Zapf A, Elben S, Wojtecki L, Tucha O, Kalbe E (2016) Screening for Cognitive Impairment in Parkinson's Disease: Improving the Diagnostic Utility of the MoCA through Subtest Weighting. PLoS One 11(7):e0159318. https://doi.org/10.1371/journal.pone.0159318
3. Sulzer P, Becker S, Maetzler W, Kalbe E, van Nueten L, Timmers M, Machetanz G, Streffer J, Salvadore G, Scholz E, Tkaczynska Z, Brockmann K, Berg D, Liepelt-Scarfone I (2018) Validation of a novel Montreal Cognitive Assessment scoring algorithm in non-demented Parkinson's disease patients. J Neurol 265(9):1976-1984. https://doi.org/10.1007/s00415-018-8942-4
4. Skorvanek M, Goldman JG, Jahanshahi M, Marras C, Rektorova I, Schmand B, van Duijn E, Goetz CG, Weintraub D, Stebbins GT, Martinez-Martin P; members of the MDS Rating Scales Review Committee (2018) Global scales for cognitive screening in Parkinson's disease: Critique and recommendations. Mov Disord 33(2):208-218. https://doi.org/10.1002/mds.27233
5. Pirogovsky E, Schiehser DM, Litvan I, Obtera KM, Burke MM, Lessig SL, Song DD, Liu L, Filoteo JV (2014) The utility of the Mattis Dementia Rating Scale in Parkinson's disease mild cognitive impairment. Parkinsonism Relat Disord 20(6):627-631. https://doi.org/10.1016/j.parkreldis.2014.03.010
6. Llebaria G, Pagonabarraga J, Kulisevsky J, García-Sánchez C, Pascual-Sedano B, Gironell A, Martínez-Corral M (2008) Cut-off score of the Mattis Dementia Rating Scale for screening dementia in Parkinson's disease. Mov Disord 23(11):1546-1550. https://doi.org/10.1002/mds.22173
7. Verbaan D, Jeukens-Visser M, Van Laar T, van Rooden SM, Van Zwet EW, Marinus J, van Hilten JJ (2011) SCOPA-cognition cutoff value for detection of Parkinson's disease dementia. Mov Disord 26(10):1881-1886. https://doi.org/10.1002/mds.23750
8. Lucza T, Ascherman Z, Kovács M, Makkos A, Harmat M, Juhász A, Janszky J, Komoly S, Kovács N, Dorn K, Karádi K (2018) Comparing Sensitivity and Specificity of Addenbrooke's Cognitive Examination-I, III and Mini-Addenbrooke's Cognitive Examination in Parkinson's Disease. Behav Neurol 2018:5932028. https://doi.org/10.1155/2018/5932028
9. Federico A, Maier A, Vianello G, Mapelli D, Trentin M, Zanette G, Picelli A, Gandolfi M, Tamburin S (2015) Screening for Mild Cognitive Impairment in Parkinson's Disease: Comparison of the Italian Versions of Three Neuropsychological Tests. Parkinsons Dis 2015:681976. https://doi.org/10.1155/2015/681976
10. Kalbe E, Calabrese P, Kohn N, Hilker R, Riedel O, Wittchen HU, Dodel R, Otto J, Ebersbach G, Kessler J (2008) Screening for cognitive deficits in Parkinson's disease with the Parkinson neuropsychometric dementia assessment (PANDA) instrument. Parkinsonism Relat Disord 14(2):93-101. https://doi.org/10.1016/j.parkreldis.2007.06.008
11. Riedel O, Klotsche J, Spottke A, Deuschl G, Förstl H, Henn F, Heuser I, Oertel W, Reichmann H, Riederer P, Trenkwalder C, Dodel R, Wittchen HU (2008) Cognitive impairment in 873 patients with idiopathic Parkinson's disease. Results from the German Study on Epidemiology of Parkinson's Disease with Dementia (GEPAD). J Neurol 255(2):255-264. https://doi.org/10.1007/s00415-008-0720-2
12. Gasser AI, Calabrese P, Kalbe E, Kessler J, Rossier P (2016) Cognitive screening in Parkinson's disease: Comparison of the Parkinson Neuropsychometric Dementia Assessment (PANDA) with 3 other short scales. Rev Neurol (Paris) 172(2):138-145. https://doi.org/ 10.1016/j.neurol.2015.08.004
13. Pignatti R, Bertella L, Scarpina F, Mauro A, Portolani E, Calabrese P (2014) Italian version of the Parkinson Neuropsychometric Dementia Assessment (PANDA): a useful instrument to detect cognitive impairments in Parkinson's Disease. J Parkinsons Dis 4(2):151-160. https://doi.org/10.3233/JPD-130270
14. Barton B, Grabli D, Bernard B, Czernecki V, Goldman JG, Stebbins G, Dubois B, Goetz CG (2012) Clinical validation of Movement Disorder Society-recommended diagnostic criteria for Parkinson's disease with dementia. Mov Disord 27(2):248-253. https://doi.org/ 10.1002/mds.24059
15. Antonini A, Tesei S, Zecchinelli A, Barone P, De Gaspari D, Canesi M, Sacilotto G, Meucci N, Mariani C, Pezzoli G (2006) Randomized study of sertraline and low-dose amitriptyline in patients with Parkinson's disease and depression: effect on quality of life. Mov Disord 21(8):1119-1122. https://doi.org/10.1002/mds.20895
16. Barone P, Scarzella L, Marconi R, Antonini A, Morgante L, Bracco F, Zappia M, Musch B; Depression/Parkinson Italian Study Group (2006) Pramipexole versus sertraline in the treatment of depression in Parkinson's disease: a national multicenter parallel-group randomized study. J Neurol 253(5):601-607. https://doi.org/10.1007/s00415-006-0067-5
17. da Silva TM, Munhoz RP, Alvarez C, Naliwaiko K, Kiss A, Andreatini R, Ferraz AC (2008) Depression in Parkinson's disease: a double-blind, randomized, placebo-controlled pilot study of omega-3 fatty-acid supplementation. J Affect Disord 111(2-3):351-359. https://doi.org/10.1016/j.jad.2008.03.008
18. Devos D, Dujardin K, Poirot I, Moreau C, Cottencin O, Thomas P, Destée A, Bordet R, Defebvre L (2008) Comparison of desipramine and citalopram treatments for depression in Parkinson's disease: a double-blind, randomized, placebo-controlled study. Mov Disord 23(6):850-857. https://doi.org/10.1002/mds.21966
19. Werneck AL, Rosso AL, Vincent MB (2009) The use of an antagonist 5-HT2a/c for depression and motor function in Parkinson' disease. Arq Neuropsiquiatr 67(2B):407-412. https://doi.org/10.1590/s0004-282x2009000300007
20. Menza M, Dobkin RD, Marin H, Mark MH, Gara M, Buyske S, Bienfait K, Dicke A (2009) A controlled trial of antidepressants in patients with Parkinson disease and depression. Neurology 72(10):886-892. https://doi.org/10.1212/01.wnl.0000336340.89821.b3
21. Menza M, Dobkin RD, Marin H, Mark MH, Gara M, Buyske S, Bienfait K, Dicke A (2009) The impact of treatment of depression on quality of life, disability and relapse in patients with Parkinson's disease. Mov Disord 24(9):1325-1332. https://doi.org/10.1002/mds.22586
22. Barone P, Poewe W, Albrecht S, Debieuvre C, Massey D, Rascol O, Tolosa E, Weintraub D (2010) Pramipexole for the treatment of depressive symptoms in patients with Parkinson's disease: a randomised, double-blind, placebo-controlled trial. Lancet Neurol 9(6):573-580. https://doi.org/10.1016/S1474-4422(10)70106-X
23. Richard IH, McDermott MP, Kurlan R, Lyness JM, Como PG, Pearson N, Factor SA, Juncos J, Serrano Ramos C, Brodsky M, Manning C, Marsh L, Shulman L, Fernandez HH, Black KJ, Panisset M, Christine CW, Jiang W, Singer C, Horn S, Pfeiffer R, Rottenberg D, Slevin J, Elmer L, Press D, Hyson HC, McDonald W; SAD-PD Study Group (2012) A randomized, double-blind, placebo-controlled trial of antidepressants in Parkinson disease. Neurology 78(16):1229-1236. https://doi.org/10.1212/WNL.0b013e3182516244
24. Meloni M, Puligheddu M, Sanna F, Cannas A, Farris R, Tronci E, Figorilli M, Defazio G, Carta M (2020) Efficacy and safety of 5-Hydroxytryptophan on levodopa-induced motor complications in Parkinson's disease: A preliminary finding. J Neurol Sci 415:116869. https://doi.org/10.1016/j.jns.2020.116869
25. Deutsche Gesellschaft für Neurologie e.V. (DGN) (2016) S3-Leitlinie Idiopathisches Parkinson-Syndrom. https://register.awmf.org/assets/guidelines/030-010k_S3_Parkinson_Syndrome_Idiopathisch_2016-06-abgelaufen.pdf. Accessed 9 April 2024
26. Seppi K, Weintraub D, Coelho M et al. The Movement Disorder Society Evidence-Based Medicine Review Update: Treatments for the non-motor symptoms of Parkinson's disease. Mov Disord 26 (Suppl 3):S42-S80.
